# Commissioners’ views and experiences of implementing virtual wards in Integrated Care Systems in England: a longitudinal qualitative study using the Consolidated Framework for Implementation Research (CFIR)

**DOI:** 10.1186/s12913-026-14740-7

**Published:** 2026-05-27

**Authors:** Laura J. McGowan, Beth Nichol, Jan Lecouturier, Helen Banks, Raenhha Dhami, Michael P. Kelly, Falko F. Sniehotta, Fiona Graham

**Affiliations:** 1 NIHR Policy Research Unit Behavioural and Social Sciences – Population Health Sciences Institute, Faculty of Medical Sciences, Newcastle University, Newcastle upon Tyne, UK; 2 School of Communities and Education, Northumbria University, Newcastle upon Tyne, UK; 3 Centre for Preventive Medicine and Digital Health (CPD), Division for Prevention of Cardiovascular and Metabolic Diseases, Medical Faculty Mannheim, Heidelberg University, Mannheim, Germany; 4 Department of Public Health and Primary Care, University of Cambridge, Cambridge, UK; 5 Centre of Preventive Medicine and Digital Health (CPD), Division of Public Health, Social and Preventive Medicine, Medical Faculty Mannheim, Heidelberg University, Mannheim, Germany

**Keywords:** Virtual wards, Hospital at home, Implementation science, Consolidated framework for implementation research, Framework analysis, Patient-centred care

## Abstract

**Background:**

Virtual wards (VWs) have been rapidly adopted within the National Health Service (NHS) in England, showing promise for reducing hospital admissions and length of hospital stays. Additionally, VWs are reportedly highly acceptable to patients and may be preferable to physical wards (PWs) in some instances. However, little research exists on how to optimise the implementation of VWs from a strategic perspective. The current study aimed to investigate commissioners’ perceptions and experiences of implementing VWs in integrated care systems (ICSs) in England, with a particular focus on changes and continuities in implementation influences over time.

**Methods:**

A longitudinal qualitative design was adopted. Semi-structured interviews were conducted via Teams with commissioners involved in the implementation of VWs at two timepoints (TP) six months apart; TP1 (*n* = 20) and TP2 (*n* = 14). Interviews focused on perceptions and opinions of the VWs programme, including barriers and facilitators to successful implementation. Data were analysed using the Consolidated Framework for Implementation Research (CFIR) in line with the Framework Method. The CFIR comprises five domains: Innovation, Outer Setting, Inner Setting, Individuals, and Implementation Process.

**Results:**

Implementation progress diverged across sites at follow-up, with some scaling back due to challenges while others expanded their VW programs. On a domain level, Inner Setting (ICS) constructs appeared most influential on implementation over time, with constructs in the Individuals domain seemingly becoming less important. The main barriers to implementation included: limited staff capacity, recruitment issues, and inefficient communication and interoperability of systems (Inner Setting); uncertainty around long-term funding (Outer Setting); inequalities in patients’ home circumstances and lack of buy-in from clinicians (Individuals); and lack of detailed implementation planning (Implementation processes). Key facilitators included: building on existing similar services (Innovation); collaborative working (Inner Setting); and national guidance (Outer Setting) with flexibility to adapt to local contexts (Implementation Process). Commissioners perceived patient acceptability to be generally high, with a reported preference for receiving care at home.

**Conclusions:**

Buy-in from clinicians, collaboration, detailed advanced planning on an ICS level, and sustainable funding are particularly important for implementation of VWs. To avoid generating inequalities, tailored additional support should be provided to disadvantaged patients. Directions for future research includes exploring the perspectives of clinicians.

**Supplementary Information:**

The online version contains supplementary material available at 10.1186/s12913-026-14740-7.

## Introduction

A rise in non-communicable diseases [1], which often co-occur [2], and population ageing have caused a growth in rates of hospital admissions and readmissions [3, 4] of patients with complex needs [5]. In England, the COVID-19 pandemic increased the average length of a hospital stay such that healthcare services were forced to tighten their criteria for hospital admission, indicating insufficient capacity of hospital beds [6]. Concurrently, staff capacity within healthcare settings is becoming more limited and often insufficient to meet the demand [7]. This challenge is alarmingly apparent in the publicly funded National Health Service (NHS) in England, creating uncertainties about its sustainability [8]. Innovations are therefore needed to optimise the available resources. Virtual wards (VWs) are led and managed by hospitals to provide acute care to patients within their own home [9], where they would otherwise be admitted to a physical ward (PW). Thus, VWs are implemented to prevent admissions onto a PW (known as step-up models), or to support patients to return home sooner after admission to a PW (known as step-down models) [9]. Although the extent of the utilisation of technology by VWs varies widely from not at all [10, 11] to a complete reliance [12], VWs typically utilise one or multiple remote monitoring technologies [13]. Indeed, the NHS defines VWs as technology enabled [14]. VWs are often conflated with Hospital at Home (HaH) services, which is a more established concept describing the provision of multidisciplinary short-term community care within the home [9]. Consequently, NHS England (NHSE) now refer to VWs as HaH [15], although HaH models typically do not integrate remote monitoring [13].

VWs presented a clear benefit for infection control during the COVID-19 pandemic [19] and have since expanded to other care pathways. In 2022, NHSE instructed all Integrated Care Systems (ICSs – partnerships of organisations that come together to plan and deliver joined up health and care services) to implement VWs in their geographic regions, supported by their Integrated Care Boards (ICB; organisations with strategic and financial responsibility for the planning and delivery of health services) [15]. The mandate encouraged the implementation of VWs nationally whilst allowing ICSs to tailor pathway models to fit their local needs [16], with respiratory and frailty pathways being the initial focus [16, 17].

Existing evidence has found VWs to be safe [18] and equal or favourable to PWs in improving clinical outcomes including mortality [9]. Robust evidence to support the effectiveness of VWs is still needed, although the available evidence indicates a reduced length of stay for step-down VWs [9]. Additionally, compared to inpatient care on PWs, models of VWs with varying degrees of use of technology show reduced rates of hospital readmission [18], although the certainty of evidence about reduced readmissions is low [9, 18]. Although robust evidence to evaluate cost-effectiveness of VWs is lacking [9, 18], one study indicated a high probability of cost-effectiveness for patients with COPD exacerbations [19]. Furthermore, there is evidence to suggest that patients show a higher acceptability towards VWs compared to PWs [9]. Thus, VWs could help to alleviate the pressures on the NHS through providing an alternative to PWs that is highly acceptable to patients [20–22]. Such models of care have also shown promise for patient care in international settings [23, 24].

Existing literature on VWs and HaH models of care reports that many of the challenges and enablers relate to the increased involvement of patients in their own care, including patient engagement [9, 25], education [9, 25], self-management and self-monitoring [9, 26], and worries about inclusivity and equal access due to differing home circumstances [26]. Further barriers include healthcare professionals adjusting to new ways of working including roles and responsibilities [24], ensuring sustainable funding [21, 22, 27], and technological difficulties including interoperability of digital solutions [24]. Meanwhile, trust and communication between all stakeholders [20, 22] and engaged and experienced leadership and management [20, 27] have been identified as key facilitators. However, most of the existing qualitative literature on the implementation of HaH and VWs are specific to COVID-19 [20, 25, 26], and the remaining literature either focuses on delivery of face-to-face care at home that is not technology enabled [22], or a single remote monitoring system [27]. In practice, VWs are complex as the use of remote monitoring is combined with differing degrees of face-to-face care, telephone consultations, input of carers, and other remote monitoring systems. Thus, an in-depth exploration of technology enabled VWs is needed to understand the barriers and facilitators to implementing this complex innovation.

Furthermore, existing research regarding the implementation of VWs rarely includes the input of stakeholders involved in leadership and management despite these roles being key to enforcing the national mandate for VWs within ICSs [28]. Specifically, there is a paucity of literature on the views and experiences of VW commissioners [9]. Within the NHS, “commissioning” refers to the process of assessing needs, planning and prioritizing, and purchasing and monitoring health services and is undertaken by those in senior leadership roles [29]. Commissioners in the NHS have a crucial role in garnering buy-in from staff including healthcare professionals (HCPs) who deliver VWs, encouraging partnership working to integrate services, and ultimately implementing VWs at scale [10].

Theoretical approaches are essential for understanding the potential mechanisms through which an intervention may work [30]. This study draws upon the Consolidated Framework for Implementation Research (CFIR) [31], a ‘meta-theoretical’ framework collating concepts from multiple implementation science theories into one overarching typology. The CFIR is therefore comprehensive in scope, and can be applied to better understand the implementation of different types of innovations across varying micro and macro level contexts. The updated version of the CFIR was used in this study [32], which comprises the following five domains: (1) Innovation – the ‘thing’ to be implemented; (2) Outer Setting – the wider environment that the inner setting exists within; (3) Inner Setting – the ultimate setting within which the innovation is implemented; (4) Individuals – the thoughts and feelings of specific actors involved in implementation of the innovation; (5) Implementation Process – the activities and strategies to embed the innovation. These domains each have multiple associated constructs and have been adapted to the VW context for this study. For example, the overarching environment of NHSE (Outer Setting) surrounds the conditions of individual ICSs (Inner Setting). In addition, the CFIR has been extended to include outcomes of implementation [33]. Adapted domain definitions and associated constructs are shown in Supplementary Material 1.

### Aims and objectives

Given the paucity of evidence of the implementation of VWs from a strategic perspective, we aimed to investigate how VWs can be optimised in practice through exploring commissioners’ perceptions of the adoption of VWs in ICSs in England, including the barriers and enablers to successful implementation. Building on inductive cross-sectional findings reported from an earlier phase of the research [28], the objective of the current paper was to compare changes and continuities in implementation influences over time using the CFIR.

## Methods

In line with Medical Research Council guidelines, a qualitative design was selected as most appropriate [30, 34]. Incorporating qualitative inquiry into intervention evaluation offers a contextualised understanding of key experiences, facilitators, and barriers to implementation across various settings. Reporting of this study adhered to the consolidated criteria for reporting qualitative research (COREQ) checklist [32] (see Supplementary Material 2).

### Study design

The study had a qualitative, longitudinal design, whereby participants were interviewed at two timepoints (timepoint one; TP1, and timepoint two; TP2) across a six-month interval. A longitudinal design was selected as appropriate to monitor the progress of implementation and allow for reflections on how barriers and facilitators changed over time. A six‑month interval was chosen because commissioners interviewed in Autumn 2022 indicated that follow‑up in Spring 2023, after navigating winter pressures, would enable meaningful reflection on implementation progress and how barriers and facilitators had evolved. Overarching inductive insights from data analysis at TP1 have been published elsewhere [28]. The current paper focuses on findings directly relating to the CFIR domains, with comparisons made between TP1 and TP2.

### Ethical approval

The study was conducted in accordance with the Declaration of Helsinki and received approval from Newcastle University’s Ethics Committee on July 28, 2022 (Ref: 23846/2022). Informed consent was audio recorded verbally at the beginning of each interview.

### Wider stakeholder engagement

NHSE colleagues involved in behavioural science strategy (and who had been tasked by the NHSE national team to understand the barriers and drivers to VWs) were involved in the conceptualisation of the study, gave direction on areas of policy relevance during data collection and analysis, assisted with sense checking of findings, and provided feedback on outputs. Additionally, a Patient and Public Involvement and Engagement (PPIE) representative was a member of the research team. Although she did not have direct personal experience of receiving care via a VW, she is a cancer survivor with extensive experience of NHS services. She contributed a patient perspective throughout the study, including feedback on the development of the interview topic guide, the conduct of the research, and the meaningfulness, clarity, and interpretation of the analysis.

### Sample

Participants were individuals in commissioning roles within ICSs, of which there are 42 across England. Commissioners within NHS settings are involved in senior leadership activities, such as planning and purchasing healthcare services [35]; all participants in this study were directly involved in the planning, implementation, and/or monitoring of VW programmes. Participants were sampled from a range of geographical regions and at varying stages of implementation of VWs to enable comparisons across contexts to be made. Some ICSs were at early stages of planning and strategizing for VWs and did not yet have their VWs up and running; ICSs in more advanced stages of implementation were already implementing VWs in practice, with some having procured enabling technologies. Typically, organisations at more advanced stages of implementation already had HaH or digitally enabled care models that could be adapted, whereas those at earlier stages were developing their VW plans from the ground up. See Supplementary Material 3 for more detailed description of the represented VWs.

Sample size at TP1 was reached following principles of data adequacy [36, 37], relating to the quality of the available data in terms of depth, variation, and amount, rather than sample size alone. For the current study, appraisal of data adequacy was based on the quality and variation of data in relation to the research question and objectives. Ongoing discussions between the research team were held throughout data collection, with data adequacy appraised at 20 participants at TP1 (for further details see: [28]). Sample size at TP2 was determined by convenience sampling as the proportion of participants from TP1 who were willing and able to participate in a second interview six months later.

### Recruitment

NHSE collaborators on the project assisted in initially identifying eligible individuals based on their professional knowledge and connections and obtained permissions to share their contact details with the research team. The research team then invited them to participate via email, with prospective participants receiving an information sheet detailing the study requirements and data protection measures. Snowball sampling was also used, with those who participated in interviews asked to identify other eligible participants. Participants provided their consent to be contacted to arrange a second interview at TP2.

### Data collection

Data collection took place between September 2022 and January 2023 at TP1, and April and June 2023 at TP2. One-to-one, semi-structured interviews were conducted online via Microsoft Teams by a member of the research team (LM or FG).

The topic guides (see the Supplementary Material 4 and 5) were applied flexibly in accordance with participant responses. To allow for conversational flow and the exploration of aspects that were most important to the participant, questions within the topic guides were not directly based on the CFIR; instead, broader open-ended questions were constructed in relation to the research aims, with questions narrowing in focus as issues of importance were explored in more detail (i.e. the ‘funnel approach’; [38]), as per published guidance [39]. Topic guides were initially developed by the research team and subsequently reviewed by a PPIE representative and NHSE collaborators, with amendments made accordingly. For example, amendments included adding a definition of VWs as technology enabled, exploring the perceived sustainability of VWs, and including probes about carers. Across both timepoints, topic guides generally focused on participants’ perceptions and opinions of the VWs programme and barriers and facilitators to successful implementation. The topic guide at TP2 was adapted from the original topic guide at TP1 to include questions prompting participants to reflect more on advancements in the implementation of VWs since TP1, including working with new technology, adaptations to planned and actual implementation, and perceptions of success.

Interviewers collected field notes throughout the interviews to assist with analysis. Video recordings captured through Microsoft Teams were converted to audio files, transcribed verbatim, and anonymised.

### Data analysis

Both datasets (TP1 and TP2) were analysed separately using the Framework method [40] before being compared. Analysis comprised the following main stages: **(1)** Familiarisation – transcripts were read and re-read and audio recordings listened to, noting key issues and idea. **(2)** Indexing – transcripts were deductively coded to the CFIR [32]. In accordance with guidance for applying the CFIR [31, 32], each construct was first operationalised for application within the current study, and some constructs were added or amended to ensure relevance (see Supplementary Material 1). Also, to ensure comprehensive coding of the data, outcomes were also coded using the Outcomes Addendum to the CFIR [33]. The indexing stage was facilitated through use of NVivo Version 14 software. **(3)** Charting – a matrix for each CFIR domain was generated in Microsoft Word, whereby the data for each sub-domain from each participant was summarised in table format, including relevant quotes. **(4)** Interpreting – matrices were reviewed and summaries from TP1 and TP2 were compared to identify similarities and difference across time.

The findings presented summarise the key constructs identified under each CFIR domain. Similar and related relevant constructs are grouped for conciseness, and the specific CFIR construct labelled in brackets. Much of the data under the Outcomes Addendum was related to the previous domains. Thus, the Outcomes Addendum is not included separately in the discussion of findings below (instead see Supplementary Material 6 for the Outcomes data). Where present, differences between timepoint one (TP1) and timepoint two (TP2) are discussed. A summary of the data coded under each CFIR construct is presented in Supplementary Material 6.

### Rigour, trustworthiness, and reflexivity

Various steps were taken to ensure rigour and trustworthiness of data collection, analysis, and interpretation. Interviews were conducted by two different researchers (LM and FG). Both researchers were female research associates (PhD credentials) experienced in conducting qualitative research, and not known to participants. Neither researcher had previously investigated VWs so did not possess prior assumptions or expectations. Further, multiple researchers were involved at various stages of analysis (LM, FG, JL, RD, HB, BN). Researchers kept notes throughout data collection and analysis processes, and regular meeting were held with the wider research team, who collectively had a range of disciplinary expertise (e.g. psychology, sociology, behavioural science, and public health), throughout the project to discuss early findings, compare coding (and adjust according to any discrepancies) and consider interpretations of data. Discussions also involved a PPIE representative and NHSE colleagues. Together, these processes ensured findings were considered from multiple perspectives and ensured relevance to public and policy interests.

## Results

### Participant characteristics and context

Twenty participants were interviewed at TP1 (September 2022 – January 2023), and 14 at TP2 (April 2023 – June 2023). For context, initial implementation plans for VWs were submitted in the Spring of 2022. One new participant was included at TP2 who had not participated at TP1, as they were identified by one of the original participants (who had since changed roles) as having a stronger role within the VWs programme at TP2. Seven of the interviewees from TP1 did not respond to follow-up interview requests at TP2, or were otherwise unavailable. Interviews lasted between 30 and 80 min at TP1 and 33 and 73 min at TP2.

At TP1, participants (*n* = 20) were aged between 34 and 60 years (Mean = 48, Standard Deviation = 7), mostly female (64%), spread across a range of managerial roles but mainly working within an NHS Trust (60%), with a range of between 3 months and 15 years of experience in their commissioning roles. Full details of participant characteristics are shown in Table 1.

**Fig. 1:**
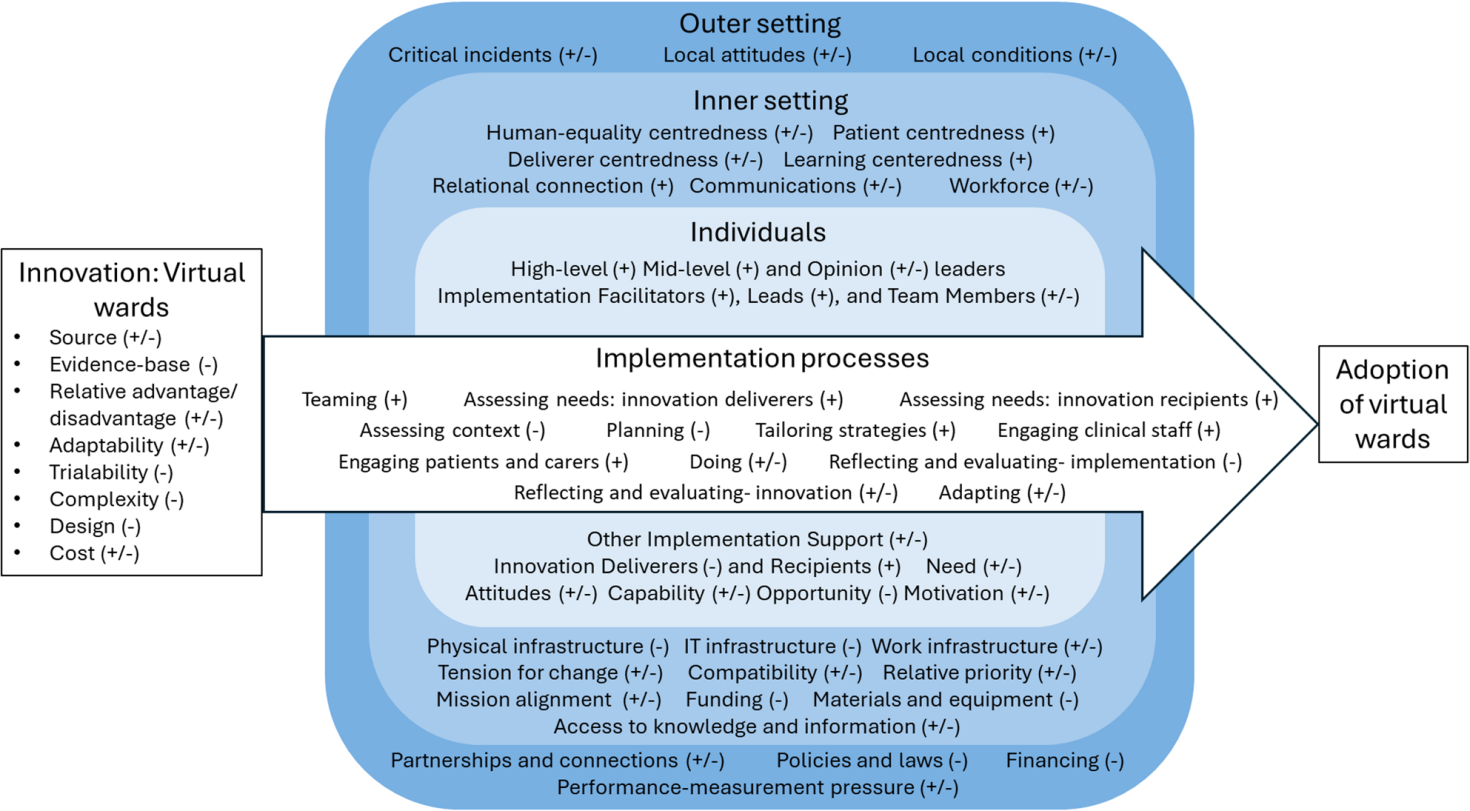
CFIR constructs identified as a barrier (-), facilitator (+), or both (+/-) to the implementation of VWs

Table 2 demonstrates prioritisation of CFIR domains in relation to the implementation of VWs, and the main barriers and facilitators within each CFIR domain (see Supplementary Material 6 for full matrix summaries). Figure 1 displays the relevant constructs to implementing VWs, and whether they were identified as a barrier, facilitator, or both.

**Table 1:** Participant characteristics at TP1

Characteristic	Total (*N* = 20)
Age in Years	
Median (Range)	48 (34–60)
Gender	
Female Male	16 (64%) 4 (36%)
Professional role	
Executive/director Programme lead/manager Senior managerial Clinical lead	6 6 6 2
Employer	5
NHS ICS NHS Trust AHSN	12 5
Geographic location	
North-East and Yorkshire North-West Midlands South-West	12 4 1 3

Notes: ICS = Integrated Care System; AHSN = Academic Health Sciences Network

**Table 2:** Key barriers and facilitators to the implementation of VWs identified for each CFIR domain

CFIR Domain	Barriers	Facilitators	TP1		TP2	
			No. codes	Rank	No. codes	Rank
Innovation	• Scepticism towards the motivation of NHSE as focused on short-term cost savings and pressures to integrate technology, not patient benefit • Limited evidence base to support VWs in terms of effectiveness, safety, responsibility, and litigation (and subsequent lack of endorsement by NICE and NIHR)	• VWs as an alternative not a replacement to PWs (allowing patient choice) • Technology utilised only if needed	624	1	178	3
Outer Setting	• Overbearing top-down pressures of unrealistic expectations • Short-term, fixed funding • Lack of time to appropriately pilot VWs • Uncertainties around allocation of costs between NHSE and ICSs	• Top-down pressures to implement VWs • National guidance which allows for adaptation to the local context (increases ownership) • COVID pandemic (encouraged use of digital solutions) • Demand for person-centred care	203	6	101	5
Inner Setting	• Limited staff capacity • Difficulty recruiting (highly skilled) staff • Interoperability issues with IT infrastructure • Lack of integration across teams • Limited efficiency of communication pathways • Lack of long-term funding • Implementation of VWs not prioritised (as VWs not developed enough to be a viable alternative to PWs) • Uncertainty of ICS funding • Inequity of access to training and guidance	• Shared learning (facilitated by NHS Futures platform) • Integrated health and social care • Utilising existing capacity, systems, teams, knowledge, and digital technologies from similar services • Collaboration and relationships between all stakeholders (facilitated by networks and meetings) • Initiatives to encourage equity amongst patients (e.g. allocated funding) • Need for change due to limited capacity of PWs • Blended approach of remote monitoring and clinician contact • VWs align with other agendas (e.g. care at home, integrated care, and person-centred care) • ‘Digital onboarding’ provided to patients • Maturity of ICS	416	3	272	1
Individuals	• Acute care staff as more hesitant and worried about reduced oversight of patients • Reluctance to change • Perception that PWs are in patients’ best interests • Belief that VWs will not reduce demands on PWs • Perception that VWs are extra work • Reduced intrinsic motivation due to top-down pressures • Worries of expected negative outcomes (e.g. intervention-generated inequalities, heightened risk)	• Utilisation of experienced and highly skilled community care staff who are tolerable to risk • Strategic leadership • Buy-in from clinicians as opinion leaders • Project management • Enthusiastic ‘champions’ of VWs • Technological support for staff and patients • Readiness of patients (in relation to autonomy and digital solutions in healthcare) • Clinician trust in community workforce • Motivation to improve patient experience	261	4	58	6
Implementation Processes	• Lack of detailed, long-term central and local planning • Minimal problem solving in advance of implementation	• Collective effort • Responding to feedback • Assessment of patient suitability for VWs • Innovative recruitment strategies to utilise existing workforce • Strategies to address interoperability issues (e.g. allocated staff capacity) • Gradual and considered implementation (particularly where similar services are not already established)	534	2	255	2
Outcomes	• Mixed acceptability from clinicians • Mixed acceptability amongst staff and carers • VWs not appropriate for all wards e.g. end of life care	• General high patient acceptability of VWs • Perceived and observed benefits to patients, deliverers, and key-decision makers	257	5	172	4

### Innovation domain: VWs

#### Innovation source

Across timepoints, participants expressed some scepticism about the sustainability of innovations by NHSE due to the short-termism of fixed term funding, with the VWs innovation being no exception.

*And a tendency that I’ve seen before in the NHS. So*,* we have a good idea and people give it a year and then they decide it hasn’t worked in which case the funding’s pulled*,* it gets dismantled*,* and we do something else.* (P05, TP1)

#### Innovation evidence-base

At both timepoints participants reported a lack of a robust evidence base to draw upon to support VWs specifically in relation to effectiveness, safety, responsibility, and litigation, creating a reluctance and uncertainty towards VWs, particularly amongst clinicians.

*I think generally the evidence base isn’t there yet in the UK*,* because these services are relatively new.* (P06, TP1)

*What we’ve heard is that it seems to be the right thing*,* but there isn’t a great deal of evidence yet to support that. That’s the hesitancy that I’ve heard from clinicians. *(P16, TP2)

#### Innovation relative advantage/disadvantage

Participants perceived many advantages of VWs, including: quicker patient reablement and recovery, reduced risk of hospital acquired infection, flexibility for those with caring responsibilities (including pets) to stay at home, and improved job satisfaction of HCPs (e.g. opportunities for staff development and new ways of working). They also thought there could be improved patient flow in hospitals, reduced ambulance queues and waiting times in A&E, some alleviation of winter pressures, as well as reduced carbon emission (through less travel to and from hospitals).

*it isn’t going to fix everything but if it manages some of these patients differently*,* safely and more effectively to reduce those ambulance queues*,* to reduce those A&E waits*,* then I think that’s a big driver for a lot of clinicians *(P05, TP1)

These positive views were seemingly reinforced at TP2 as participants directly observed often unexpected benefits, such as tech-enabled remote monitoring providing patients with reassurance. Participants also reported that clinicians had a higher level of oversight of VW patients than they originally anticipated.

*it has given them a lot of reassurance that they are being monitored […] we’re not calling them multiple times a day just to say*,* “Everything is still alright*,*” sort of thing*,* because we’re able to see that […] which I think is probably best for everyone because*,* I suppose*,* if the patient is at home*,* part of them feeling like they’re getting better is that they’re not having constant check-ins* (P04, TP2)

However, worries remained about potential of intervention-generated inequalities given patient-level differences in home circumstances, ability to access and use the relevant technology, and increased pressure on unpaid carers.

*It’s making sure that the patient’s personal circumstances are responded to in terms of that digital poverty and inclusion*,* balanced with*,* you know*,* those isolated communities that may be more at risk. And I think people are considering it*,* but whether we are at the point yet where we’re tackling it and ensuring that there’s no further disadvantage*,* I think we’re too early. (P03*,* TP2)*

Further, VWs were considered to provide less opportunity for HCPs to *‘get your eyes on somebody*’ (P13, TP1), meaning safeguarding issues may be less likely detected. Some participants generally felt that VWs carried a higher risk in terms of opportunity for error.

*It’s endless the amount of different things that could go wrong.* (P02, TP2)

Further disadvantages discussed were the risk of losing patient trust, and the preference for some elderly or frailer patients to be in hospital due to being lonely at home:

*the trust and the relationships that need to be built up to support people in environments like this […] If any of those things contradict what they’ve been told or not what they’ve been told*,* then you’ve lost really about trying to support them in a confident way*,* in a trustworthy way and they won’t feel safe. We’ll end up back into traditional settings.* (P15, TP2)

*what they recognise in frailty is that […] one of the things that made the patient reluctant to go home was that they were actually a bit lonely.* (P18, TP2)

At TP2, participants were still unsure whether VWs were to be used to provide additional capacity, or intended to be a direct replacement for a hospital bed (as described in further detail in our previous publication; [28]).

#### Innovation adaptability

The adoption of VWs for respiratory illness was generally through adapting or expanding existing services. However, there were no such existing services for patients with frailty. Frailty was acknowledged as a more complex health issue to treat via VW. There appeared to be a general preference to avoid remote monitoring and use of technology, primarily due to concerns that the dexterity required for their use may exceed the capabilities of patients living with frailty. The desire for a standardised model of VWs across localities to maintain quality and consistency appeared to lessen from TP1 to TP2, as participants increasingly saw the benefit of being able to adapt the service to the assets, needs, and challenges within each locality.

*If you look at one acute provider*,* the services that they offer are all slightly different*,* and the staffing pressures that they have are all slightly different*,* so you can’t have a one size fits all of virtual wards across the patch.* (P02, TP2)

#### Innovation trialability

The paucity of examples of piloting and trialling VWs mainly related to their constituent technological elements rather than VWs themselves. Participants discussed a lack of time to appropriately trial VWs during a pilot period before their implementation.

#### Innovation complexity

VWs were perceived as highly complex in terms of: the variation of constituent technological elements and the skill and knowledge required of both service providers and users to operate them, the scope of the wide variation of circumstances and patients who utilise them and multiple comorbidities and needs within patients, the integration required with a myriad of existing systems and services, and the partnership working that involves coordination between numerous organisations. One participant offered a solution of a *hub model* to provide a central contact for navigation across VWs and counter the challenges of communication within such complex systems.

*They need one central contact*,* that will be*,* “Who do I ring for virtual wards?” So that sort of hub model for the remote monitoring could help with that navigation around who is who and who the right contact person is.* (P12, TP2)

### Outer setting: England

#### Critical incidents

The COVID-19 pandemic facilitated the uptake of VWs as HCPs became more acclimatised to remote working and digital solutions following initial reluctance. Contrastingly, staff strikes further reduced the limited staff capacity to use VWs.

*we’ve noticed our occupancy*,* when there’s been strikes*,* suffers*,* because if clinicians are covering other clinicians*,* then they’re not recruiting patients on to the virtual wards.* (P20, TP2)

#### Partnerships and connections

Participants valued sharing learning and collaborating at TP1 and networked with other trusts and the ICB commissioning support unit and learnt about best practice through NHSE. Connections and networks had developed at TP2 to include networking between clinical leads, acute trusts, and community service providers.

#### Policies and laws

Participants discussed difficulty accessing the relevant information related to VW policy. Particularly at TP2, participants discussed feeling overwhelmed at the constant revisions to policy and standards around VWs, and called for *‘less changing goalposts’* (P06, TP2) to allow them to *‘just*,* kind of*,* let us get on with it’* (P13, TP2). Across both timepoints, a movement towards positive changes to policy such as data sharing agreements to enable the implementation of VWs was yet to materialise.

#### Financing

Funding from NHSE gradually declined and seemingly ceased after two years; £200 m was provided for the first financial year of 2022/23 and matched the second year, but after that no national funding was referenced by participants. Thus, across timepoints, participants felt that the implementation of VWs by NHSE was hasty and represented short-term and short-sighted thinking towards financial cost-savings, creating unrealistic expectations and pressures.

*We’ve got a culture that doesn’t see things out as long as it should. Then we start to say*,* ‘Oh*,* it didn’t work.* (P07, TP2)

#### External pressure

External pressure related to both performance-measurement and implementation. Participants were somewhat ambivalent about a heavy reliance on top-down pressures to implement VWs; whilst some recognised that it accelerated implementation, others felt that it could be unhelpful and paternalistic, that it did not allow for a period of adjustment and piloting, and that the targets and milestones were unrealistic and not informed by practice.

*So it’s kind of a bit of a double-edged sword*,* really. In one respect*,* you’ve got to take your time and do everything*,* and dot all the Is and cross all the Ts. […] But ultimately*,* we don’t live in an ideal world*,* do we? So that’s just how it is. In the NHS*,* the pressures that we’re under all the time are phenomenal. […] But actually*,* you do make it happen. You get on with it. So in some respects*,* it keeps you going. But it’s definitely a challenge.* (P13, TP1)

### Inner setting: ICSs

#### Infrastructure

Larger ICSs could present a barrier to the implementation of VWs due to increased complexity (Structural Characteristics: Physical infrastructure):

*That has taken an absolute age*,* but it takes […] a lot of commitment of time because you’re having the same- you’re having conversations at place and then having them at across place and then having to get your clinicians to agree across place*,* your commissioners to agree across place*,* your community providers*,* your system people*,* your IG*,* the whole lot*,* across three separate places* (P08, TP2)

The interoperability of IT systems (discussed in our previous publication: [28]) remained a major barrier to implementation of VWs at TP2 (Structural Characteristics: Information technology infrastructure). Another key barrier at TP2 was limited staff capacity, in part driven by difficulty recruiting staff (Work infrastructure). Integrated care, including situation of VWs within existing capacity and teams and appropriate utilisation of the highly skilled acute and community care workforce, was not yet fully established but was noted to facilitate implementation of VWs (Work infrastructure).

*it’s just been that things always take a lot longer to implement. I think it’s been working through how we do that sort of regionally*,* as well*,* with the new ICS landscape* (P14, TP1)

#### Culture

Concerns related to intervention-generated inequalities demonstrate that participants valued equity and equality of patients and worked to achieve this (Human-equality centredness). It was acknowledged that patient experience of VWs largely depended on their home circumstances, including availability of food and heating, space, social support, cleanliness, access to technology, costs of additional technology, and rurality of residence. At TP2, initiatives were discussed to address equality-related concerns raised at TP1, including allocated funding for those without stable home circumstances. However, reports were mixed about whether health inequalities were a core part of discussions around VWs or an afterthought.

*I haven’t heard a lot about that health inequity lens*,* to be very honest. I think that’s because of where we are in people getting their head around just trying to get these things up and running. […] It’s certainly not excluded*,* but certainly not the top of the agenda at the moment* (P15, TP2)

Person centred care was central to participants, who also noted clinicians as advocates for patient choice (Patient-centredness). At TP2, the adjustments to VWs that participants described were primarily to improve patient experience, such as a blended approach of remote monitoring and clinical input and expanding relevant outcomes to include quality indicators of patient experience (Patient-centredness).

#### Relative priority

Insufficient funding for the sustainable implementation of VWs appeared to lead to a fragmented approach to delivery, particularly with respect to digital healthcare, thus hindering the development of VWs as a viable model of care. This perception was reported across both TP1 and TP2 and was attributed to prioritisation of hospital care over primary and community care.

*We’re trying to piecemeal the [VWs] offer together. We need a fundamental shift really in the prioritisation of resource and funding […] Don’t focus all your attention on the hospital environment. Focus your attention on community services*,* primary care and social care […] Get serious about resourcing tech*,* digital interoperability and shared records […] really accelerate it because it’s critical for some of this* (P15, TP2)

Participants reported that this fragmented approach led to clinicians addressing rising admission rates through a more reactive strategy—referred to as “firefighting” (P04, TP1)—by reverting to PW admissions. This preference suggested that PWs were viewed as a more immediate and practical solution, given that VWs had not yet been fully established or optimised, thus limiting their operational effectiveness. This was particularly evident at times of increased system pressure, such as winter months where admissions increased. Although it was acknowledged at TP1 that VW care should be prioritised at such times of high system pressure, it was reported at TP2 that this planned prioritisation often did not translate into practice.

*Due to the pressure the whole urgent emergency care system is under and the fact that*,* as we head into this winter when we think*,* it’s going to get worse*,* there’s been a massive push and priority to have this and urgent community response in place. It’s been a big priority for everybody to make sure this is up and running* (P09, TP1)

*The programme of work that’s clinically led is very*,* very tough especially in the areas of respiratory and frailty when numbers of inpatients increase during the winter months and those specialties*,* specifically. That meant that our clinicians were often pulled from doing virtual ward work or doing virtual ward development work to walk the wards and do medical outlying wards and additional beds open*,* etc’* (P08, TP2)

#### Mission alignment

Alignment of VWs to the agendas of the ICSs (which was influenced by political agendas) was mostly discussed at TP1. VWs were noted to align with interrelated agendas around providing care closer to home, integrated care between primary, secondary and community care, preventing and reducing hospital admissions, and person-centred and personalised care. However, to align with the agenda to reduce health inequalities, adjustments to VWs were needed, for example providing technology or providing paper-based alternatives. It was noted that VWs should be applied flexibly to patient needs and local contexts rather than implemented indiscriminately.

#### Funding

Uncertainty around the allocation of costs between NHSE and individual ICSs combined with short-term national funding resulted in recruitment difficulties and scaling back of implementation plans at TP2. More established ICS were more likely to be able to match national funding, creating variation across ICSs and leading to potential inequity of access across regions.

*So it’s been joint funded this year by the ICB and NHSE and then next year it’s ICBs only. And ICBs are newly formed and they’re not- I don’t think they’ve fully found themselves yet. And so making these big decisions about finances there’s a lot of uncertainty*,* so then clinical teams are*,* like*,* “Well*,* how are we going to do this if we don’t know where the funding’s coming from? Because our directors of finance won’t let us recruit to posts unless there’s recurrent funding.”* (P05, TP2)

Participants described a predicament of being required to demonstrate effectiveness of VWs to ensure funding from the ICS but requiring funding to be able to operate efficiently and effectively.

#### Materials and equipment

Difficulty with the procurement of technology was anticipated at TP1 and reported by most participants at TP2, including disagreements about which technology to use, variable experiences with contracts with technology companies, and inconsistency across VWs.

*there’s just been variability in the uptake in those across our patch. If we had been more co-ordinated at the outset*,* we would’ve agreed on one or two providers of this kind of technology*,* and then providers could’ve used from a framework. *(P01, TP2)

#### Workforce

Concerns were frequent around the limited capacity of highly skilled specialist staff who are able to provide the complex community care required for VWs, particularly for frailty. Participants were unsure whether such staff would be newly recruited, voicing concerns about the small pool from which to recruit new staff, or whether they would be existing staff, which raised concerns that staff would be removed from other services. Subsequently, implementation plans were sometimes scaled back at TP2.

*You’re pinching from Paul to pay Peter. So*,* yes*,* it’s a very*,* very difficult and challenging issue*,* I think. And it’s going to be- and it has been flagged as one of the risks around the delivery of the virtual wards is having that workforce* (P07, TP1)

In contrast, only one participant described sufficient staff capacity to deliver VWs, which was achieved through redistributing existing NHS staff.

#### Access to knowledge and information

Staff appreciated the NHS Futures platform for accessing training and guidance, although at TP2 it became apparent that not all staff were provided with the same opportunity for knowledge acquisition in the absence of centralised training guidelines or routes. Relatedly, at TP2 participants discussed a need for centralised guidance on technology procurement, staffing, and support whilst allowing for flexibility locally.

### Individuals

#### Leaders

High-level leaders were important in providing strategic system-wide oversight of the planning and delivery of VWs and provided top-down pressure through monitoring data and funding. Support in capability and capacity from high-level leaders was rare but highly valued. Positive attitudes of senior clinicians were highly respected and subsequently buy-in from clinicians was discussed as essential for the successful implementation of VWs, which was described as varied but mainly lacking (Opinion leaders).

*If you haven’t got clinical buy-in to do this from the start*,* it just won’t happen* (P02, TP1)

Participants discussed both top-down (e.g. support from management) and bottom-up (e.g. enthusiastic individuals) facilitators of VWs (Opinion leaders).

#### Implementation support

Participants described multiple implementation leads with distinct strengths – project managers to provide practical support, frontline champions to encourage adoption of VWs, and clinical leads as key influencers to facilitate the collaboration required for progress of VWs.

VWs were exemplary of integrated care as their implementation required collaboration between primary, secondary, community, and social care providers, which remained both a priority and a challenge across timepoints (Implementation Team Members).

#### Characteristics

Participants generally showed high levels of enthusiasm towards VWs as a new way of working and expressed feelings of pride at TP2 for the progress made at implementing VWs, although top-down pressures to accelerate implementation of VWs decreased their intrinsic motivation and enthusiasm (Motivation).

However, participants also described scepticism shared with some deliverers about the motivations behind implementing VWs and their potential to reduce demand on services, thus participants perceived acceptability amongst key stakeholders to be mixed, particularly amongst clinicians (Motivation). Participants reported accounts of varied clinician acceptability depending on their beliefs about the potential of technological solutions, perceptions of VWs as extra work, motivations to provide care in the community as opposed to PWs, tolerance to risk, enthusiasm towards new ways of working, and flexibility (Motivation). Participants also felt that community care staff were more tolerant to risk compared to acute care staff, however felt that acute care staff had accrued more confidence at TP2 as they realised the competency of community care (Capability). On the contrary, participants reported patient acceptability to be high, as one participant discussed a wider movement towards patients possessing more autonomy over their own care through access to digital solutions, indicating a readiness of patients to engage in VWs (Motivation). Participants described competing priorities for staff that was perceived to be more immediate than developing VWs (Opportunity).

### Implementation processes

#### Teaming

Participants recognised the importance of collaboration and coordination at TP1 and were enacting it by TP2, citing examples including building on existing relationships between hospitals and community teams, utilising existing specialist networks, multidisciplinary working groups, and monthly steering group meetings. Similarly, participants noted the value of broadening out collaborations to include local authorities and social care and described team working with a wide variety of stakeholders at TP2 including remote monitoring providers. It was evident across time that collaboration to deliver VWs had become ‘*united’* (P13, TP2).

*So*,* there’s a lot of connectedness*,* collective leadership. A lot of co-production*,* you know*,* really trying to do this collegiately* (P03, TP2)

#### Assessing needs: innovation deliverers

A minority of participants described formal methods of gathering information on the needs and preferences of deliverers, for example through surveys or workshops with GPs and clinicians, respectively. More often, participants discussed feedback on the needs relating to implementation of VWs from deliverers without specifying how it was collected, indicating more informal processes. For example, participants discussed feedback from deliverers on limited staff capacity which guided an investment into greater capacity and capability in community care.

*I think we’ve tried to ask clinicians what they really want* (P18, TP2)

#### Assessing needs: innovation recipients

At TP1 and to a greater extent at TP2, some participants described collecting feedback on the patient experience of VWs including through informal discussions, real-time questionnaires, and case studies. At TP2 participants also described including carers in shared decision making around admission onto VWs, and assessing individual suitability for VWs through home visits.

#### Planning

Some level of planning, including an outline of the project timeline and goals in terms of number of VW beds, was required to secure initial funding for VWs and thus was enforced by NHSE. However, there was a distinct lack of long-term detailed planning, impacted by uncertainty around finance and funding and a lack of knowledge concerning the processes involved in implementing VWs as a novel innovation. At both timepoints, some participants discussed a lack of critical thinking about how the logistics and operations of VWs would work in context (Assessing context).

*Probably we didn’t speak to people as well as we should have before we set this off.* (P15, TP2)

#### Tailoring strategies

Variation was observed both in how the VWs mandate was enacted by ICSs and how strategies to implementation were amended according to unanticipated barriers. Specific examples included adopting innovative recruitment strategies such as *‘retire and return’* and *‘flexible working’* (P06, TP1) to address the barrier of recruitment, and allocating specific staff capacity to address interoperability and procurement issues.

#### Engaging stakeholders

Participants described collaborative activities to foster enthusiasm and increase buy-in from deliverers, including workshops, training modules, a community of practice, and networks (Engaging clinical staff). In some cases, feedback from patients had informed subsequent implementation and delivery of VWs (Engaging patients and carers).

#### Doing

At TP2, participants noted that a *‘soft launch’* or *‘phased’* approach to implementation, where patient numbers on VWs were initially kept low, enabled better identification of issues, thus ensuring adaptability of the innovation in a safe and meaningful way.

*it has given us the chance to learn and resolve some of those issues*,* whereas*,* if we’d been at 20-patient capacity immediately and still identifying some of the connectivity and interoperability-type issues that we have done*,* there’d have been much more of an impact of those issues* (P04, TP2)

However, many participants utilised existing systems and pathways to implement VWs, thus *‘baby steps’* (P15, TP2) were less relevant and appropriate.

#### Reflecting and evaluating

Participants named numerous indicators of successful implementation. For example, some participants discussed plans to measure numbers of patients referred to VWs, use of digital solutions utilised by VWs, referrals into VWs (as an indication of established pathways and buy-in from clinicians), longevity of the project, to what extent VWs was embedded into *‘business as usual’* (P16, TP2), and staff capacity. However, no detail was provided on how such information was collected.

Across timepoints, participants expressed the need for a funded, systematic, and sustained evaluation of the costs and benefits of VWs.

*So*,* there’ll be those real hard measures around bed numbers and admissions and so on as well as some of the qualitative measures around patient experience and how they found the experience and so on. So*,* I think that will obviously be a piece of work that will have to go on […] in terms of really understanding the benefits.* (P18, TP2)

## Discussion

This longitudinal qualitative study investigated commissioners’ views and experiences of implementing VWs in ICSs in England, using the CFIR. On a domain level, characteristics of VWs (Innovation) appeared to become less relevant over time, although participants felt that their scepticism about the motivation of VWs had been validated at TP2. On the contrary, the ICS environment and processes (Inner Setting) appeared to become more relevant to implementation of VWs across time. Whilst interoperability issues with IT infrastructure remained a key challenge, the ramifications of short-term national funding and thus reliance on continued funding from ICSs became apparent at TP2 through recruitment difficulties and scaling back of implementation. Strategies to implement VWs (Implementation Processes) remained relevant across time, although at TP2 participants demonstrated a stronger preference for a gradual and flexible approach to implementation that accounted for local needs and existing infrastructure. Also, at TP2 participants described a myriad of strategies that were developed on a local level to overcome unexpected challenges to implementation. Whilst top-down pressures to implement VWs remained stable across time (Outer Setting), Outer Setting remained relatively irrelevant to implementation. Individual action (Individuals) became even less important to the implementation of VWs as the importance of collaboration and collective buy-in became more valued and embedded. Integrating siloed ways of working across social care and primary, secondary, and community healthcare remained a key challenge. However, at TP2, relationships, connections, and networks between teams had developed which fostered trust. Finally, outcomes were in many ways more positive than expected, including high patient acceptability and the ease of ‘on-boarding’ patients onto IT infrastructure (Outcomes).

The current study identified CFIR domains Inner Setting and Implementation Processes to remain relevant across time with Inner Setting becoming increasingly relevant, whilst Outer Setting was, and Innovation and Individuals increasingly became, relatively irrelevant across timepoints. These findings reflect existing literature investigating the implementation of other digital services. For example, existing literature on telehealth services identifies Inner Setting to be most commonly reported as an influential domain [41]. Similarly, an assessment of a remote monitoring technology using the CFIR identified Inner Setting and Implementation Processes to be particularly influential for successful implementation [27]. Although the aforementioned study [27] also identified the Individuals Domain to also be particularly influential, this was driven by the lack of buy-in from clinicians and may also be attributable to the reduced complexity of a single remote monitoring service compared to VWs.

In accordance with the existing literature on VWs, this study identified that cohesion between teams and collective action [42] was essential to implementing VWs [43, 44]. Collaboration allowed for challenges in implementation to be addressed with solutions that were framed within the local context. However, to optimise shared learning, it would be beneficial to share such strategies with other ICSs through the variety of communication pathways outlined by participants, including NHS futures, strategy meetings, and networks to develop a ‘community of practice’ [43]. Furthermore, the mixed acceptability of VWs amongst clinicians remained a key barrier to implementation over time as clinicians were highly influential over other stakeholders; a finding replicated in other studies of similar innovations [27, 43]. On the contrary, a key facilitator of the implementation of VWs was their alignment to wider national [45, 46] and global [47] agendas for person-centred and integrated care, as VWs provide an option for care at home, encourage patients to self-manage, and require collaboration between teams within and beyond healthcare. However, VWs align with person-centred care only if patients are provided with a choice of care and are not mandated onto VWs. One key finding not yet identified within previous VWs research was a lack of implementation planning. Although the ability of ICSs to develop solutions to challenges in implementation as implementation progressed was a facilitator, it also indicated a lack of detailed planning and problem solving a priori. Consequently, the implementation of VWs was iterative and thus could be inefficient, particularly regarding IT infrastructure. However, the current study identified that implementation plans should be specific to local needs and context, given that VWs are a highly complex service implemented in a context of unique local conditions and existing services.

Several key findings from this study align with broader trends observed in healthcare initiatives, particularly within the NHS. For example, this study identified an inherent challenge of short-term funding for VWs as sufficient long-term funding was required to implement VWs at scale, which was necessary to demonstrate sustainability and cost-effectiveness; described as a discrepancy between short run and long run efficiency [48]. Also, this study identified a greater focus on the outcomes of VWs compared to implementation outcomes, despite success of an innovation being strongly impacted by the quality of its implementation [49, 50]. Teams were expected to both implement and demonstrate positive outcomes of VWs simultaneously, instead of engaging in adaptation and implementation prior to a formal evaluation [51]. This approach is a barrier to the success of many other NHS initiatives as high pressures on the NHS does not allow for long-term funding and planning [52]. Short-term funding can prevent sustainable and scaled implementation [53], which reduces the likelihood of achieving cost-effective outcomes [50].

## Implications and suggestions for future research

The findings of the current study highlight four key implications for the optimisation of VWs. First, intervention is needed to address the key challenge in the implementation of VWs as a of lack of buy-in from clinicians. One solution proffered by one participant was to amend the VWs model so that the lead is an allied health professional, for example, rather than a doctor. Also, the CFIR can be mapped onto implementation strategies outlined by the Expert Recommendations for Implementing Change (ERIC) compilation [54]. Utilising the ERIC taxonomy matching tool [55], strategies to address clinicians as opinion leaders include “*identify and prepare champions*”, through informing clinicians about their influence over the successful implementation of VWs, and inform Local Opinion Leaders (identified in the CFIR as individuals with informal influence on the attitudes and behaviours of others), through providing clear information about the rationale behind VWs and their emerging positive outcomes. Future research should focus on clinicians to explore determinants of acceptability and mechanisms of influence on other stakeholders, to inform interventions to increase acceptability amongst clinicians or the development of alternative models of implementation of VWs that do not require clinicians to lead them. 

Second, detailed a priori planning is recommended for further implementation of VWs. Planning at local level should assess contextual barriers and facilitators to implementation and build from existing systems and services where possible. Plans should include integration with existing IT infrastructure, development of, and establishing, clear communications pathways, and integration of services. Indeed, the aforementioned study of barriers and facilitators to the implementation of a remote monitoring app also identified a lack of planning as a barrier to implementation, and recommended adapting the CFIR [27] and using The Quality Implementation Framework [56] to map contextual barriers and implementation strategy, respectively. Relatedly, the current study found that top-down pressures decreased motivation to implement VWs, including amongst clinicians. Thus, involvement of local stakeholders in planning of VWs would help to increase ownership [57] which is crucial for ensuring sustainability [58–60]. 

Third, to optimise implementation of VWs and improve the likelihood of success and sustainability, greater practical top-down support is needed through continued funding and staff capacity, facilitated by improved planning. Last, a frequent concern was the potential of VWs to generate inequalities, known as intervention-generated inequalities [61], both on an ICS and individual level. As such, proactive measures are needed not only to prevent the exacerbation of these disparities, but also to ensure that VWs actively contribute to reducing existing health inequalities. On an individual level, there is a need for an adapted and tailored approach to the implementation of VWs that provides more support to specific patients, and at TP2 many sites organically developed strategies to address this. However, such tailored support should also occur at an ICS level to ensure that the implementation of VWs within less mature ICSs are sustainable once national funding is withdrawn.

### Strengths and limitations

Longitudinal qualitative studies are rare in implementation research [62, 63], yet they are key to understanding implementation process and particularly sustainability [63]. Thus, a key strength of this study was that the longitudinal design allowed for the assessment of how implementation had developed, and which barriers and facilitators became more or less relevant as implementation progressed. However, given that this study identified collective action as essential, it would have been relevant and valuable to sample a range of stakeholders [64, 65], including the patient voice which is lacking in implementation research [64] including on VWs [9]. Furthermore, even less is known about the experiences of both formal and informal carers [9, 66]. Whilst the current sample of commissioners offered a broad overarching oversight of the implementation of VWs from individuals who acted as mediators between the outer (NHSE) and inner (individual ICSs) setting, sampling of a single stakeholder group may have missed key experiences and perspectives relevant to implementation [64]. For example, perceptions of patient attitudes were tenuous and far removed.

## Conclusions

Implemented appropriately, VWs are exemplary of integrated and person-centred care and the importance of collective action for implementation of a novel innovation. However, to ensure that VWs become an equally valued alternative to PWs by deliverers, sustained and long-term funding is required to support planning, strategic leadership, staff capacity, relationship building, operation and integration of IT infrastructure, monitoring and measurement of outcomes, and equity for all patients. Without this, it is unlikely that VWs will be demonstrated to be implementable and cost-effective. Although further national leadership and guidance is required, it is essential that local teams can take ownership of implementation plans and amend them to local assets and needs. It is equally important that deliverers are provided with the time and space to share such learning with each other across localities, to utilise the valuable knowledge and strategies that emerge locally. Finally, clinician buy-in is essential for ensuring engagement of other stakeholders.

## Electronic Supplementary Material

Below is the link to the electronic supplementary material.

Supplementary Material 1: Operationalised CFIR Framework

Supplementary Material 2: COREQ Checklist

Supplementary Material 3: Description of VWs

Supplementary Material 4: Topic Guide TP1

Supplementary Material 5: Topic Guide TP2

Supplementary Material 6: Summary CFIR Matrices

## Data Availability

Data is provided within the manuscript or supplementary information files.
