# Supplementary material for: Commissioners’ views and experiences of implementing virtual wards in Integrated Care Systems in England: a longitudinal qualitative study using the Consolidated Framework for Implementation Research (CFIR)
Source: BMC Health Serv Res. 2026 May 27;26:1027. doi: 10.1186/s12913-026-14740-7 (PMC13404884; doi:10.1186/s12913-026-14740-7)
Supplement: Supplementary file 1 — Supplementary Material 1: Operationalised CFIR Framework [file 12913_2026_14740_MOESM1_ESM.docx]

| **I. INNOVATION DOMAIN Virtual ward model- enabled by technology [Document the innovation being implemented, e.g., innovation type, innovation core vs. adaptable components, using a published reporting guideline. Distinguish the innovation (the “thing” that continues when implementation is complete) from the implementation process and strategies used to implement the innovation (activities that end after implementation is complete).]** | |
| --- | --- |
| **Construct Name** | **Construct Definition** *The degree to which:* |
| A. Virtual ward model(s) Source | The group that developed and/or visibly sponsored use of 'virtual ward models' is reputable, credible, and/or trustable. i.e. NHS England |
| B. Virtual ward model Evidence-Base | The Virtual ward model(s) have a robust evidence supporting its effectiveness. Examples of VW models include hospital @ home models, remote monitoring, telehealth/digital care hubs. |
| C. Virtual ward model(s) Advantage/Disadvantage | Virtual ward model(s) better than other care delivery models/current practice. i.e. benefits to patients, hospital flow etc. Also code disadvantages here e.g. more work for existing staff, additional staff training requirements, requires patient 'onboarding' etc. |
| D. Virtual ward model(s) Adaptability | The virtual wards model(s) can be modified, tailored, or refined to fit local context or needs. Code examples of where they talk about developing an agnostic virtual ward that can be applied/adapted for different conditions/patient groups. |
| E. Virtual ward model(s) Trialability | The virtual ward model(s) can be tested or piloted on a small scale and undone. Code instances where interviewees talk about pilot tests they have done such as with AHSN, models of technologies. |
| F. Virtual ward model(s) Complexity | The virtual ward model(s) are complicated, which may be reflected by their scope and/or the nature and number of connections and steps. Code descriptions of the different elements/components/teams the virtual ward (care delivery model) have. e.g. where they describe it being complex as it requires the involvement of social care, primary care, community care and acute trust staff in the delivery, requires integrated patient medical records, tech and software for staff and patients etc. |
| G. Virtual ward model(s) Design (NHSE mandate and definition of VW) | The VW model(s) are well designed and packaged, including how it is assembled, bundled, and presented. Use this to capture reflections about mandate and the description of virtual wards presented by NHSE, language around virtual wards. |
| H. Virtual ward model(s) Cost | The purchase and operating costs of the tech-enabled VW models are affordable. Capture concerns expressed about the cost of the tech and resources required to deliver the innovation. Discussions around potential cost-savings of VW way would go in VW advantage |
| I. Description of virtual ward | Descriptions of virtual wards (including plans)- care pathways, health conditions, tech, dashboards, devices |
| **II. OUTER SETTING DOMAIN Outer Setting: The setting in which the Inner Setting exists, e.g. England.** | |
| **Construct Name** | **Construct Definition** *The degree to which:* |
| A. Critical Incidents | Large-scale and/or unanticipated events disrupt implementation VW programme and/or delivery of care by VW. E.g. Pandemic, NHS cyber attack, NHS strikes |
| B. Local Attitudes | Sociocultural values (e.g., shared responsibility in helping recipients) and beliefs (e.g., convictions about the worthiness of recipients) encourage the Outer Setting to support implementation and/or delivery of the innovation. General consensus amongst English citizens that NHS should provide care to all, and that patients should receive the best quality care available. Patients should have the option to be care for at home? (Public perception of the benefits/disadvantage of VW should go in I. INNOVATION DOMAIN- C. Relative Advantage/Disadvantage) |
| C. Local Conditions | Economic, environmental, political, and/or technological conditions enable the England as a nation to support implementation and/or delivery of virtual ward model(s) in the NHS. Code references to the outer setting- England- Recovery from covid, impact winter will have on hospital pressures and VW implementation, cost of living crisis- e.g. people unable to heat their homes (variability in patients individual circumstances nationally). Rurality |
| D. Partnerships & Connections | The ICS is networked with external entities, including academic affiliations, and professional organization networks. ICS is networked with academic health sciences network, voluntary sectors? Connections and networks across ICSs |
| E. National guidance and policies | Legislation, regulations, professional group guidelines and recommendations, or accreditation standards support implementation and/or delivery of the VW models. Reflections on the usefulness of NHSE National platforms and communications from NHSE Future learn/Shared communities of practice - Central platforms and guidelines produced by NHSE to support implementation. Endorsement from Royal societies (Royal college of nursing/ general practitioners/surgeons etc). ?NICE guidelines |
| F. Financing | Funding from NHSE available to implement and/or deliver the virtual ward mandate. Reflections upon funding structures and streams. |
| G. External Pressure | External pressures drive implementation and/or delivery of the VWs. Note: Use this construct to capture themes related to External Pressures that are not included in the subconstructs below. e.g. Tech companies, Government |
| 1. Societal Pressure | Mass media campaigns, advocacy groups, or social movements or protests drive implementation and/or delivery of the innovation. There could be something positive/ negative in the press about virtual wards that could make the job of implementation more difficult. If reports of waiting times/pressures on beds etc. are featured in the external press then I would code here. [If they are internal, i.e. within their own ICS are a driver would code at III. INNER SETTING DOMAIN-Tension for Change |
| 2. Market Pressure | Competing with and/or imitating peer entities drives implementation and/or delivery of the innovation. Potentially references to private healthcare |
| 3. Performance-Measurement Pressure and implementation/delivery speed | Quality or benchmarking metrics or established service goals drive implementation and/or delivery of the innovation. Reference to both ICS and NHSE performance metrics can be coded here. Time pressure to demonstrate impact/success, pressure to implement at pace without time to reflect, short-termism, reporting requirements |
| **III. INNER SETTING DOMAIN Inner Setting: The setting in which the innovation is implemented, integrated care systems (ICS).This includes integrated care partnerships (ICB- Local Authorities), ICBs, Local Authorities (social care and public health functions as well as other vital services for local people and businesses.) Place-based partnerships (NHS, local councils, community and voluntary organisations, local residents, people who use services,) Provider collaboratives (acute trusts, community care, general practice (PCNs, GP federations), emergency care (ambulance services).** | |
| **Construct Name** | **Construct Definition** *The degree to which:* |
| *Note:* | *Constructs A – D exist in the Inner Setting regardless of implementation and/or delivery of the innovation, i.e.,* ***they are persistent general characteristics of the Inner Setting.*** |
| A. Structural Characteristics | Infrastructure components support functional performance of the Inner Setting. Note: Use this construct to capture themes related to Structural Characteristics that are not included in the subconstructs below. E.g. maturity, and size of ICS. |
| 1. Physical Infrastructure | Layout and configuration of space and other tangible material features support functional performance of the ICS. Capture any reference to the proximity of sites within an ICS, shared premises for example. |
| 2. Information Technology Infrastructure | Technological systems for tele-communication, electronic documentation, and data storage, management, reporting, and analysis support functional performance of the ICS. This more about the infrastructure that supports tech enabled VW/HAH rather than the tech that they buy-in that forms part of the intervention. May include references to existing patient medical record systems, e.g SystemOne, Nerve Centre, interoperability with data collection platforms, dashboard [References to devices such as wearables to go in description of innovation. |
| 3. Work Infrastructure | Organization of tasks and responsibilities within and between individuals and teams, and general staffing levels, support functional performance of the ICS. General staffing levels/issues more broadly in the ICS that are not specific to the delivery of virtual wards. |
| B. Relational Connections | There are high quality formal and informal relationships, networks, and teams within and across Inner Setting boundaries (e.g., structural, professional). Working relationships with colleagues- not specific to virtual wards necessarily |
| C. Communications | There are high quality formal and informal information sharing practices within and across ICS boundaries (e.g., structural, professional). Including with other ICSs, IT teams, patients, clinicians. |
| D. Culture | There are shared values, beliefs, and norms across the ICS. Note: Use this construct to capture themes related to Culture that are not included in the subconstructs below. How we do things/do things best/would like to do things |
| 1. Human Equality-Centeredness | There are shared values, beliefs, and norms about the inherent equal worth and value of all human beings. General ethos around reducing health inequalities. |
| 2. Patient-Centeredness | There are shared values, beliefs, and norms around caring, supporting, and addressing the needs and welfare of patients. General ethos of putting the patients first- broader than the virtual ward agenda |
| 3. Deliverer (staff)-Centeredness | There are shared values, beliefs, and norms around caring, supporting, and addressing the needs and welfare of deliverers. Ethos of ensuring staff job satisfaction, findings the best ways of working, opportunities for staff development rather than skills and capability. [Code skills and capability of staff at IV. INDIVIDUALS DOMAIN- Capability] |
| 4. Learning-Centeredness | There are shared values, beliefs, and norms around psychological safety, continual improvement, and using data to inform practice. May not come up but potentially any reference around the culture of striving to improve and learn from doing- again not necessarily VW specific. |
| *Note:* | ***Constructs E – K are specific to the implementation and/or delivery of VW*** |
| E. Tension for Change | The current situation is intolerable and needs to change. Include overall rationale for why VWs could be a good idea |
| F. Compatibility | The VW fits with workflows, systems, and processes. E.g. Existing Hospital at Home models/ Covid 19 remote monitoring etc. |
| G. Relative Priority | Implementing and delivering the VW is important compared to other initiatives. Maybe something mentioned in interviews with Exec/directors. |
| H. Incentive Systems | Tangible and/or intangible incentives and rewards and/or disincentives and punishments support implementation and delivery of the innovation. E.g. Monetary incentives |
| I. Mission Alignment | Implementing and delivering the innovation is in line with the overarching commitment, purpose, or goals in the Inner Setting. Inner setting ambitions to reduce numbers of patients in hospital beds/demand for bed etc. could go here. I would only code these things here if the interviewee states they are a good thing because they align with the overall goal of the NHS/ICS etc. for example patient centered care |
| J. Available Resources | Resources are available to implement and deliver the innovation. Note: Use this construct to capture themes related to Available Resources that are not included in the subconstructs below. |
| 1. Funding | Funding is available to implement and deliver the innovation. Whether the ICS can match fund/ afford to run them without the national funding. Relative wealth of the ICS, have they any other funding they could add to the NHSE funds |
| 2. Space | Physical space is available to implement and deliver the innovation. It might come up in terms of availability of space to run digital hubs/banks of computers for remote monitoring. |
| 3. Materials & Equipment | Supplies are available to implement and deliver the innovation. Include tech and procurement of tech (Staff recruitment would go under J. available resources/4. workforce below) |
| 4. Workforce | Have added new subcode 'workforce' to include references regarding difficulties recruiting adequate numbers of staff to work on the implementation and delivery of virtual wards. |
| K. Access to Knowledge & Information | Guidance and/or training is accessible to implement and deliver the innovation. Code reference to accessibility to any information, guidance, training etc here whether it's from NHSE or elsewhere (could also be from tech companies) both staff and patient training/'onboarding' |
| **IV. INDIVIDUALS DOMAIN Individuals: The roles and characteristics of individuals.** | |
| **ROLES SUBDOMAIN Project Roles: [Document the roles applicable to the project and their location in the Inner or Outer Setting.]** | |
| **Construct Name** | **Construct Definition** |
| A. High-level Leaders | Individuals with a high level of authority, including key decision-makers, executive leaders, or directors. |
| B. Mid-level Leaders | Individuals with a moderate level of authority, including leaders supervised by a high-level leader and who supervise others. |
| C. Opinion Leaders | Individuals with informal influence on the attitudes and behaviours of others. |
| D. Implementation Facilitators | Individuals with subject matter expertise who assist, coach, or support implementation. |
| E. Implementation Leads | Individuals who lead efforts to implement the innovation. |
| F. Implementation Team Members | Individuals who collaborate with and support the Implementation Leads to implement the innovation, ideally including Innovation Deliverers and Recipients. |
| G. Other Implementation Support | Individuals who support the Implementation Leads and/or Implementation Team Members to implement the innovation. |
| H. Innovation Deliverers | Individuals who are directly or indirectly delivering the innovation. |
| I. Innovation Recipients | Individuals who are directly or indirectly receiving the innovation. |
| **CHARACTERISTICS SUBDOMAIN Project Characteristics: [Document the characteristics applicable to the roles in the project based on the COM-B system or role-specific theories.]** | |
| **Construct Name** | **Construct Definition:** *The degree to which:* |
| **Innovation Deliverers** | |
| A. Need | The individual(s) has deficits related to survival, well-being, or personal fulfilment, which will be addressed by implementation and/or delivery of the innovation. |
| B. Capability | The individual(s) has interpersonal competence, knowledge, and skills to fulfil Role. |
| 1. Attitudes | The individual(s) has a positive/negative attitudes towards virtual wards/implementation process |
| C. Opportunity | The individual(s) has availability, scope, and power to fulfil Role. Include time to attend training/visit other ICS too. |
| D. Motivation | The individual(s) is committed to fulfilling Role. Barriers and resistance/reluctance |
| **Managers and directors (operational staff)** | |
| A. Need | The individual(s) has deficits related to survival, well-being, or personal fulfilment, which will be addressed by implementation and/or delivery of the innovation. |
| B. Capability | The individual(s) has interpersonal competence, knowledge, and skills to fulfil Role. |
| 1. Attitudes | The individual(s) has a positive/negative attitudes towards virtual wards/implementation process |
| C. Opportunity | The individual(s) has availability, scope, and power to fulfil Role. Include time to attend training/visit other ICS too. |
| D. Motivation | The individual(s) is committed to fulfilling Role. Barriers and resistance/reluctance |
| **Patient and carers (innovation recipients)** | |
| A. Need | The individual(s) has deficits related to survival, well-being, or personal fulfilment, which will be addressed by implementation and/or delivery of the innovation. |
| B. Capability | The individual(s) has interpersonal competence, knowledge, and skills to fulfil Role. E.g. tech skills |
| 1. Attitudes | The individual(s) has a positive/negative attitudes towards virtual wards/implementation process |
| C. Opportunity | The individual(s) has availability, scope, and power to fulfil Role. Personal circumstances household level e.g. access to Wi-Fi at home, having access to tech |
| D. Motivation | The individual(s) is committed to fulfilling Role. |
| **V. IMPLEMENTATION PROCESS DOMAIN  Implementation Process: The activities and strategies used to implement the innovation. Project Implementation Process: [Document the implementation process framework and/or activities and strategies being used to implement the innovation. Distinguish the implementation process used to implement the innovation (activities that end after implementation is complete) from the innovation (the “thing” that continues when implementation is complete).]** | |
| **Construct Name** | **Construct Definition:** *The degree to which individuals:* |
| A. Teaming | Join together, intentionally coordinating and collaborating on interdependent tasks, to implement the VW plan/VWs |
| B. Assessing Needs | Collect information about priorities, preferences, and needs of people. Note: Use this construct to capture themes related to Assessing Needs that are not included in the subconstructs below. E.g. Survey of GPs about what they need in terms to support/use virtual wards |
| 1. Innovation Deliverers | Collect information about the priorities, preferences, and needs of deliverers to guide implementation and delivery of the innovation. This is about the activity of seeking the views of deliverers and feeding that into the intervention implementation and delivery process. |
| 2. Innovation Recipients | Collect information about the priorities, preferences, and needs of recipients/patients/carers to guide implementation and delivery of the innovation. |
| C. Assessing Context | Collect information to identify and appraise barriers and facilitators to implementation and delivery of the innovation. This is about whether the ICSs are actively finding out what the barriers and enablers are. |
| D. Planning | Identify roles and responsibilities, outline specific steps and milestones, and define goals and measures for implementation success in advance. Include references to the work around care pathway development. |
| E. Tailoring Strategies | Choose and operationalize implementation strategies to address barriers, leverage facilitators, and fit context. This is about overcoming the barriers through strategies. Have interviewees strategised ways to overcome barriers they have identified? |
| F. Engaging | Attract and encourage participation in implementation planning or delivery team Note: Use this construct to capture themes related to Engaging that are not included in the subconstructs below. Focused on what's being done/can be done to **foster** buy-in and acceptability |
| 1. Clinical staff (Innovation Deliverers) | Attract and encourage deliverers to serve on the implementation planning team and/or to deliver the innovation. Have interviewees engaged clinical staff? If so how? |
| 2. Patients/Carers (Innovation Recipients) | Attract and encourage recipients to serve on the implementation planning team and/or participate in the innovation. Have interviewees engaged patients/carers? If so how? |
| G. Doing | Implement in small steps, tests, or cycles of change to trial and cumulatively optimize delivery of the innovation. Code references about how they have made changes to VW models to make them work. |
| H. Reflecting & Evaluating | Collect and discuss quantitative and qualitative information about the success of implementation. Note: Use this construct to capture themes related to Reflecting & Evaluating that are not included in the subconstructs below. => includes examples of actual evaluation work that some ICSs have done e.g. surveys/ focus groups with patients, carers staff |
| 1. Implementation | Collect and discuss quantitative and qualitive information about the success of implementation. |
| 2. Innovation | Collect and discuss quantitative and qualitative information about the success of the innovation. |
| I. Adapting | Modify the VW/Tech and/or the ICS infrastructures for optimal fit and integration into work processes. E.g. Working to address interoperability issues |
| **CFIR OUTCOMES ADDENDUM** | |
| **I. ANTECEDENT ASSESSMENTS** | |
| **Name** | **Definition** |
| A. Acceptability | The extent to which an innovation is perceived as “agreeable, palatable, or satisfactory" (Proctor, 2009). Includes patient/ staff pushback concerns about virtual wards |
| B. Appropriateness | The “perceived fit, relevance, or compatibility of the innovation […] for a given practice setting, provider, or consumer; and/or perceived fit of the innovation to address a particular issue or problem" (Proctor, 2009). => local context stuff, ideal VW patient, who for, for which health conditions and under which conditions (e.g. social network/family support), can also include tech literacy |
| C. Feasibility | The extent to which an innovation “can be successfully used or carried out within a given agency or setting" (Proctor, 2009). Includes concerns around achieving timeframes, milestones |
| D. Implementation Climate | The extent to which the Inner Setting has an implementation climate. => leadership, attitudes, resisters and champions on all levels |
| E. Implementation Readiness | The extent to which the Inner Setting is ready for implementation. => as above but includes examples of work being done Tangible and immediate indicators of organizational commitment to its decision to implement virtual wards e.g. have already identified Tech/ written standard operating procedures etc. |
| **II. IMPLEMENTATION OUTCOMES** | |
| **Name** | **Definition** |
| A. Anticipated Implementation Outcomes | Outcomes based on perceptions or measures of the likelihood of future implementation success or failure, i.e., implementation outcomes that have not yet occurred. These outcomes are forward-looking; constellations of CFIR determinants across domains predict these outcomes. Captures views about what they anticipate will be the outcome of the intervention in the longer term. E.g. care for X number of patients on a VW/close hospital beds/ revolutionise how specialist care is delivered |
| 1. Adoptability | The likelihood key decision-makers will decide to put the innovation in place/innovation deliverers will decide to deliver to innovation. |
| 2. Implementability | The likelihood the innovation will be put in place or delivered. |
| 3. Sustainability | The likelihood the innovation will be put in place or delivered over the long-term. |
| B. Actual Implementation Outcomes | Outcomes based on perceptions or measures of current (or past) implementation success or failure, i.e., implementation outcomes that have occurred. These outcomes are backward-looking; constellations of CFIR determinants across domains explain these outcomes (There be some measures of patient satisfaction they have collected) |
| 1. Adoption | The extent key decision-makers decide to put the innovation in place/innovation deliverers decide to deliver the innovation. |
| 2. Implementation | The extent the innovation is in place or being delivered. |
| 3. Sustainment | The extent the innovation is in place or being delivered over the long-term. |
| **III. INNOVATION OUTCOMES** | Outcomes that capture the success or failure of the innovation, based on the impact of the innovation on three important constituents: Innovation Recipients, Innovation Deliverers, and Key Decision-Makers. Impact is defined by: Reach ("The absolute number, proportion, and representativeness of individuals who are willing to participate in a given initiative, intervention, or program.”) x Innovation Effectiveness ("The impact of an intervention on important outcomes, including potential negative effects, quality of life, and economic outcomes.” |
| **Name** | **Definition** |
| A. Innovation Recipient Impact | Recipient Reach x Innovation Effectiveness |
| B. Innovation Deliverer Impact | Deliverer Reach x Innovation Effectiveness |
| C. Key-Decision Maker (or System) Impact | Key-Decision Maker Reach x Innovation Effectiveness |
