# Supplementary material for: Commissioners’ views and experiences of implementing virtual wards in Integrated Care Systems in England: a longitudinal qualitative study using the Consolidated Framework for Implementation Research (CFIR)
Source: BMC Health Serv Res. 2026 May 27;26:1027. doi: 10.1186/s12913-026-14740-7 (PMC13404884; doi:10.1186/s12913-026-14740-7)
Supplement: Supplementary file 5 — Supplementary Material 5: Topic Guide TP2 [file 12913_2026_14740_MOESM5_ESM.docx]

**Title of Study:** Adoption and implementation of virtual wards in Integrated Care Systems in England: A qualitative exploration of the views and experiences of Integrated Care System commissioners

**Interview Topic Guide**

*(In line with usual practice in qualitative interviewing, the questions asked are likely to evolve as interviews are conducted and key issues emerge)*

Opening statement: *Thank you again for agreeing to talk to me today. As you may recall, I am a researcher from Newcastle University.* *We are interested in better understanding the factors influencing the adoption of virtual wards across integrated care systems in England. When we last spoke, I was able to get a better understanding of the approach being taken to introduce virtual wards in your organisation. The purpose of the interview today, is find out more about how this process is going, what is working well and less well and what support may be helpful to you. Before we start, I’d like to revisit the consent form to check that you still consent to participate in this study [talk through consent form]. Please be reminded that you are free to stop the interview and withdraw your participation at any point without giving a reason.*

*Just to remind you, when I use the term ‘virtual wards’ I’m referring to virtual wards enabled by technology, i.e. the management of patients via a digital platform (e.g. using remote monitoring technology to monitor patients’ symptoms and physiological parameters). I understand that when we last spoke, some of the virtual wards services you were developing were yet to incorporate tech. I am interested in knowing more about your plans and progress with tech and whether they have changed.*

*Does that all sound ok to you? Do you have any questions before we start? Ok great, let’s get started…*

1. Can you please tell me how the implementation of virtual wards has been going since we last spoke?
   1. *Probes:* what has been working well? [insert plans/perceived effective ways of working from previous interview e.g. *Building upon existing care pathway/expanding community care teams, step-up/step-down approaches, identifying tech to support remote delivery of care, community and acute teams working together effectively, communication channels/sharing of best practice effective].*
   2. *Probes: What has been working less well? [insert (anticipated) challenges last interview e.g. Workforce shortages- recruitment of additional staff, Procurement of technology, Identification of need for tech/ health problem to be addressed by tech, development of novel care pathway,* ***governance issues, accountability structures,*** *integrated patient medical records, engaging virtual ward deliverers-staff/patients] How have challenges been overcome?*
2. Have the plans for virtual wards changed since we last spoke [month of last interview]? If so, how?
   1. *Probes: Has your job role changed at all in the process?*
   2. *Probes: Are the virtual wards being set-up for the specific conditions you described last time we spoke? [insert description of virtual wards from previous interview e.g. frailty, respiratory]? Is the main goal of the ward the same as last time we spoke? [insert description from previous interview e.g. admission avoidance, early supported discharge, both?]*
   3. *What is the scope of the virtual ward, has this changed? (monitoring v monitoring and treatment v monitoring, diagnostics & treatment)*
   4. *Do these virtual wards meet national definition/principles yet i.e. implementing as desired? If (deliberately) not, why?*
3. Can you please tell me more about the team or teams of people you are working with to organise/plan/deliver these virtual wards?
   1. *Probes: Are you still working with the same team, has this changed? Working with community care colleagues/primarily acute care/social care colleagues, patients, carers, IT/tech developers other stakeholders- Allied Health Sciences Networks?* ***How have you found this process, what has been difficult/ worked well?***
   2. *Who is leading the implementation of virtual wards in your ICS, has this changed? What attributes/qualities does these people have/lack to do this role effectively? (if enthusiasm comes up here, probe what drives this – personality, circumstantial factors etc)*
   3. *Have other organisations/individuals influenced your approach to implementing virtual wards?*
4. What technology have you been integrating into services and how? If not, why not?
   1. *Probes: How was the tech identified, how have you found the procurement process?*
   2. *How well is the tech working for staff and patients? How have you captured this?*
   3. *Probes: digital platforms to enable remote consultations or devices e.g. oximeters*, *24 hour wearables, apps).*
      1. *How was this tech decided upon?*
   4. *Delivery: Frequency of patient contact (and how contacted – face to face, remote), hours of service (12/24 hour etc), urban vs rural delivery, staff involved etc?*
5. **How do (or do you anticipate) the virtual wards will work compared to usual practice (for patients/carers/clinical practice/practitioners/trusts)?**
   1. *Probes: have you experienced the advantages for patients you anticipated (e.g. more patients staying at home, patients feel reassured)?*
   2. *Have you experienced the disadvantages for patient you anticipated (e.g. digital literacy issues)?*
   3. *How have you measured implications for health inequalities? e.g. assessment of home environment? Perceived impact on carers? Impact on clinical outcomes?*
   4. *Barriers and challenges to delivering/implementing virtual wards from HCP/trust perspectives? Personal misgivings about risk? How may affect professional identity?*
6. In your view, has the implementation of virtual wards so far been successful?
   1. *Probes: What are your measures of implementation success? What milestones are you working towards [insert milestones from previous interview] Considerations beyond the SitReps? Successful for who/what -* patients or staff, systems, patient flow, finance?
   2. *What has made it successful? Characteristics of individuals involved in implementing- local clinicians? (if enthusiasm comes up here, probe what drives this – personality, circumstantial factors etc?) Communication factors? Skills/resources? How have challenges been overcome?*
   3. *Are they working more collaboratively with organisations within the ICS? E.g. community and acute services already integrated?*
   4. *Have they different information technology infrastructures? E.g Digital care hubs, integrated patient records?*
   5. *Have they advanced information governance and structures?*
   6. *Have they more clinical engagement? Why do you think this is?*
   7. *Why has it been unsuccessful? How can any barriers be overcome? Is there anything NHS England can do differently to help?*
7. What are your personal feelings around the adoption/implementation of virtual wards?
   1. *Probes: Any emotions around this e.g. sense of achievement, important career milestone, burdensome, worried, optimistic, excited, fear around sanctions if not adopted? What worries you?*
   2. *How confident are you in the evidence for VWs? What evidence is this/where from?*
   3. *Where do you see the future of virtual wards? Potential for long-term cost-saving?*
8. What support do you need from NHSE to make VWs work?

*Closing questions*

1. Is there anything else you would like to tell us about virtual wards? Anything that we’ve not already asked you about? Or were any of our questions less relevant/important?
