# Supplementary material for: Commissioners’ views and experiences of implementing virtual wards in Integrated Care Systems in England: a longitudinal qualitative study using the Consolidated Framework for Implementation Research (CFIR)
Source: BMC Health Serv Res. 2026 May 27;26:1027. doi: 10.1186/s12913-026-14740-7 (PMC13404884; doi:10.1186/s12913-026-14740-7)
Supplement: Supplementary file 6 — Supplementary Material 6: Summary CFIR Matrices [file 12913_2026_14740_MOESM6_ESM.docx]

| **I. INNOVATION DOMAIN** | |
| --- | --- |
| A. Source | Across timepoints, the main worry expressed by participants was that the implementation of VWs by NHSE was hasty and represented short-term and short-sighted thinking towards financial cost-savings, creating unrealistic expectations and pressures. Thus, participants expressed difficulty sustaining innovations including VWs long-term after fixed-term funding from NHSE ceased. Contrastingly, one participant showed very positive views of the leadership of the NHSE, reporting them to be ‘very facilitative, very supportive, collaborative’ (P03, TP2). |
| B. Evidence-Base | Participants at TP1 reported that despite some evidence suggesting that the related service patient reablement was more effective in one’s own environment (especially for frail patients), there is a lack of a robust evidence base for to draw upon for VWs specifically in relation to effectiveness, safety, responsibility, and litigation. In turn, a lack of endorsement from NIHR or NICE due to a paucity of robust evidence created reluctance and uncertainty towards VWs particularly amongst clinicians. |
| C. Relative Advantage/ Disadvantage | Perceived advantages: patients generally heal more quickly at home and prefer to, a reduced risk of hospital acquired infection, flexibility for those with dependent including pets and caring responsibilities to stay at home, improved job satisfaction of HCPs through new opportunities for staff development and ways of working, improved patient flow in hospitals, less ambulance ques and travelling. Unexpected benefits observed at TP2 included reduced hospital admissions and length of stay, flexibility of the role for HCPs, avoiding GP and A&E visits, and a higher level of oversight of VW patients than was originally anticipated.  Perceived disadvantages: potential intervention-generated inequalities given individual differences in home circumstances and ability to access and use the relevant technology, accessibility of monitoring systems, increased pressure on carers, less oversight of patients safeguarding issues are less likely to be detected, and staff unfamiliar with working in the community may feel anxiety and stress. |
| D. Adaptability | The adaptation of respiratory VWs was largely influenced by existing services like remote monitoring, palliative care, and COVID-related initiatives. While these adaptations worked well for some areas, frailty was recognised as a more complex challenge that was harder to integrate. There was a general reluctance to rely on technology unless necessary, improving accessibility. Initially, there was interest in a standardised VW model, but over time, preferences shifted toward tailoring services to local needs. A gradual implementation approach was suggested to address emerging challenges. The level of technological integration and in-person care varied significantly across localities, though it was unclear if these differences were always driven by local needs. |
| E. Trialability | The paucity of examples of piloting and trialling VWs mainly related to their constituent technological elements rather than VWs themselves. Participants discussed a lack of time to appropriately trial VWs during a pilot period before their implementation. |
| F. Complexity | VWs were seen as highly complex due to variations in technology, the expertise required from both providers and users, and the diverse needs of patients with multiple conditions. Their successful implementation also depended on integrating with existing systems and coordinating across multiple organisations. To address these challenges, one participant suggested a ‘hub model’ to serve as a central point of contact for navigating VWs. |
| G. Design | In addition to the issues around the constituent technological elements within VWs, there was uncertainty around the aim and purpose of VWs. Again, it was expressed that the hasty implementation of VWs was interpreted by some participants as a need to meet strategic aims for integrated technology rather than for patient benefit enabled by technology. Participants felt that their assumptions at TP1 had been validated at TP2. |
| H. Cost | Discussion of operating costs was uncertain given the lack of supporting evidence and debate about whether VWs should complement or replace hospital beds mentioned above, although participants could envision some cost-savings: ‘There's the potential for savings, certainly around length of stay costs, or if you've got people who are in hospital who would predominantly have maybe a longer length of stay, the virtual ward could impact on that.’ P07, TP1 |
| **II. OUTER SETTING DOMAIN** | |
| A. Critical Incidents | Critical incidents that negatively affected implementation: Doctors and nurses strikes  Critical incidents that positively affected implementation: COVID pandemic (more acclimatised to technological solutions) |
| B. Local Attitudes | Although infrequently discussed, participants acknowledged that the NHS should provide the best quality and safest care to patients through being flexible and adaptable to their needs, which recognises that patients may prefer a PW. One participant also mentioned a culture towards empowering individuals to *‘take more responsibility and ownership of their own health’* (P13, TP2). Both reflect a demand for person centred care, which acts as a facilitator to the implementation of VWs but argues against indiscriminate roll-out across patients. |
| C. Local Conditions | It was acknowledged that the patient experience of VWs largely depended on their home circumstances including availability of food and heating, space, social support, cleanliness, access to technology, costs of additional technology, and rurality of residence. |
| D. Partnerships & Connections | Shared learning between other trusts and the ICB commissioning support unit, facilitated through NHSE. Additionally, comparison between sites was important to participants, facilitated by national webinars. Connections and networks had developed at TP2 between trust stakeholders. Although stakeholders utilised connections, there remained a disconnect between policy ad practice. |
| E. National guidance and policies | At TP1, participants discussed a need for a balance between clear guidance on a national level and the flexibility to adapt policies to local needs. Participants however discussed difficulty accessing the relevant information related to VW policy. At TP2, participants discussed feelings of overwhelm at the constant revisions to policy and standards around VWs which they perceived as overbearing and micromanaging, and called for ‘less changing goalposts’ (P06, TP2) to allow them to ‘just, kind of, let us get on with it’ (P13, TP2). Across both timepoints, discussion of movement towards positive changes to policy such as data sharing agreement to enable the implementation of VWs were yet to be materialised. |
| F. Financing | The implementation plan for VWs suffered from short-sightedness, leading to a decline in fixed-term funding that ended after two years, creating uncertainty about their sustainability. Concerns were raised about the allocation of costs and the sources of funding for various elements of VWs, highlighting the need for clearer financial responsibility. By TP2, these funding uncertainties were confirmed, resulting in delays and a reduction in the scale of implementation plans. |
| G. External Pressure | External pressures affecting VWs primarily revolved around performance measurement and implementation demands, with one instance of political pressure to decrease reliance on ambulance services. Participants expressed mixed feelings about the heavy reliance on top-down pressures to implement VWs and relieve strain on emergency and primary care services. While some acknowledged that such pressures could hasten implementation, many felt they were often unhelpful and paternalistic, not allowing time for adjustment or pilot testing. Additionally, they viewed the targets and milestones as unrealistic and not grounded in practical experience. |
| **III. INNER SETTING DOMAIN** | |
| A. Structural Characteristics | |
| 1. Physical Infrastructure | ICBs across England could differ significantly in their size, maturity, rurality, and existence of digital hubs within them, adding complexity to implementing VWs across a variety of ICSs. For example, larger ICSs reported a higher difficulty with engaging all trusts. |
| 2. Information Technology Infrastructure | The complexity of current digital systems within ICSs was a major barrier to implementation by HCPs, and some participants reported that specific capacity was required to navigate the implementation of VWs across existing IT systems within and between teams. The interoperability of IT infrastructure was highly inefficient particularly when HCPs were required to manually add data to IT systems. |
| 3. Work Infrastructure | Participants noted many aspects of the ICS infrastructure that made implementation of VWs challenging, including a weary workforce that are still adjusting to the move to ICS, limited staff capacity, uncertainty about role responsibilities, lack of integration across teams, and difficulty recruiting staff. Infrastructure that was noted to enable VWs although was not yet established included commissioning of other relevant systems to support VWs, integration of health and social care, situation of VWs within existing capacity and teams, and appropriate utilisation of the highly skilled acute and community care workforce. Thus, the main facilitator that would enable VWs is integrated care, which would in turn facilitate integration of the service into existing practice. |
| B. Relational Connections | Existing relationships between hospitals and community teams was highly valued. Building on this foundation, collaboration, collective leadership, and connectedness was reported to have improved at TP2, facilitated by specialist networks (e.g ageing well) and digital clinical safety networks. |
| C. Communications | Feedback towards communications were mixed at TP1; whilst participants valued a variety of communication channels, some participants recognised communication pathways as less developed, resulting in repeated conversations and thus limited efficiency and feelings of overwhelm. |
| D. Culture | |
| 1. Human Equality-Centeredness | The concerns related to intervention generated inequalities demonstrate that participants valued equity and equality of patients and worked to achieve this. AT TP2, initiatives were discussed to address the concerns discussed at TP1, including allocated funding for those without stable home circumstances. However, reports were mixed about whether health inequalities were a core part of discussions around VWs or an afterthought. |
| 2. Patient-Centeredness | Person centred care was central to participants, who also noted clinicians as advocates for patient choice. At TP2, the adjustments to VWs that participants described were primarily to improve patient experience, such as a blended approach of remote monitoring and clinical input and expanding relevant outcomes to include quality indicators of patient experience. |
| 3. Deliverer (staff)-Centeredness | Participants demonstrated a great respect in HCPs and were conscious of their limited capacity to adopt an innovation such as VWs. However, overall, the high pressure environment for HCPs meant that staff were overworked, had limited opportunity for learning and development, and needed time to recalibrate after disruptive events including the COVID pandemic. |
| 4. Learning-Centeredness | Regular meetings for shared learning around VWs emerged and flourished between TP1 and TP2. |
| E. Tension for Change | Participants generally recognised a unanimous bottom-up need for a new way of working at TP1. There was a clear need for change but uncertainty around VWs as the solution at TP1 but not at TP2, perhaps indicating that VWs appropriately addressed the tension for change observed at TP1. |
| F. Compatibility | Participants felt that VWs aligned with an existing movement towards digital and tele healthcare, creating a preparedness for VWs. |
| G. Relative Priority | Participants discussed the top-down mandate of the VWs programme. However, the absence of long-term funding hindered sustained implementation, resulting in a fragmented approach to digital healthcare that made VWs less viable compared to Physical Wards (PWs). This led to a "firefighting" mentality, where increased admissions were managed by PWs, viewed as a more immediate solution while VWs were still being established and operating below full efficiency due to inadequate investment in overcoming issues like interoperability. For instance, although some participants discussed using VWs to alleviate winter pressures on respiratory and frailty wards, others noted that clinicians focused on VWs were often redirected to support PWs. |
| I. Mission Alignment | Discussed the alignment of VWs with ICS agendas, influenced by political factors. VWs were seen as supportive of goals like providing care closer to home, integrating services, and reducing hospital admissions. However, to effectively address health inequalities, VWs required adjustments, such as offering technology or paper-based alternatives. Flexibility in applying VWs to meet patient needs and local contexts was emphasised over indiscriminate implementation. |
| 1. Funding | Given the barriers relating to financing from NHSE related to the distribution of funding over time rather than the total amount, the sustainability of VWs was uncertain as it relied on matched funding from ICSs which was no longer mandated at TP2, which was more likely for more established ICSs. There was also uncertainty about the allocation of costs and thus where funding for each element of VWs would come from, calling for a *‘clearer view of who pays for what’* (P17, TP1). Whilst the short-term nature of funding from NHSE was recognised at TP1, the consequences of no funding from the ICS was apparent in the uncertainty at TP2, including relating to recruitment and scaling back of implementation plans. |
| 3. Materials & Equipment | The lack of central planning and pressure to implement VWs led to varied and inefficient technology procurement, not aligned with legal guidelines and policies. This challenge was anticipated at TP1 and confirmed by most participants at TP2, who noted disagreements over technology choices, inconsistent experiences with contracts from tech companies, and variability across different VWs. |
| 4. Workforce | There were widespread concerns about the limited availability of specialist staff for VWs, especially in frailty care. Uncertainty over recruitment and the risk of pulling staff from other services led to scaled-back implementation at TP2. Only one participant reported sufficient staffing, achieved by redistributing existing NHS staff. |
| K. Access to Knowledge & Information | Staff valued the NHS Futures platform for training, but inconsistent access and a lack of centralised guidelines were noted at TP2. Participants called for standardised guidance on technology, staffing, and support while maintaining local flexibility. Training healthcare assistants for digital onboarding was seen as beneficial. |
| **IV. INDIVIDUALS DOMAIN** | |
| **ROLES SUBDOMAIN** | |
| A. High-level Leaders | High-level leaders played a key role in overseeing VWs, coordinating implementation, reporting to NHSE, and aligning VWs with broader agendas like health inequalities. They also applied top-down pressure through data monitoring and funding. While direct support for capability and capacity was rare, it was highly valued by participants. |
| B. Mid-level Leaders | Participants involved in operationalising VWs through practical oversight varied in whether they were devoted to VWs or if VWs was part responsibilities which affected their overarching interests and aims, for example contributing to the Ageing Well programme. |
| C. Opinion Leaders | Senior clinicians' support was seen as crucial for VWs, but buy-in was generally lacking. Successful implementation relied on both top-down support from management and bottom-up enthusiasm from key individuals. |
| D. Implementation Facilitators | As such, although clinical leads appeared to be the drivers of implementation of VWs, it was instead a collaboration that utilised a range of skills. |
| E. Implementation Leads | Implementation relied on project managers for support, frontline champions for adoption, and clinical leads for collaboration. |
| F. Implementation Team Members | VWs required collaboration across healthcare sectors, making integration both a priority and a challenge. |
| G. Other Implementation Support | AT TP1, wider sources of support for the implementation of VWs included the local authority, who were hesitant about the increase of home care and its repercussions for social care needs, and improvement support responsible for recording and evaluating implementation. However, at TP2, participants focused more on the value of a tech support line for staff and patients. |
| H. Innovation Deliverers | Participants described mixed acceptability of VWs among frontline deliverers due to medicolegal litigation concerns, as well as uncertainty about who would deliver VWs; namely if staff would solely be deployed to VWs or rotate with other community services Thus, the perception of innovation deliverers was unclear. |
| I. Innovation Recipients | One participant discussed a wider movement towards patients possessing more autonomy over their own care through access to digital solutions, indicating a readiness of patients to engage in VWs. |
| **CHARACTERISTICS SUBDOMAIN** | |
| **Innovation Deliverers** | |
| B. Capability | Barrier: acute care staff as more risk averse  Facilitator: community care staff as experienced and assured with tolerating risk |
| 1. Attitudes | Barrier: scepticism about the motivations for and potential of VWs  Facilitator: VWs as offering care at home |
| C. Opportunity | Barrier: Competing priorities perceived to be more urgent |
| D. Motivation | Facilitator: VWs could increase job satisfaction |
| **Managers and directors (operational staff)** | |
| B. Capability | Barrier: requires clinician leads to trust staff who they may not know  Facilitator: established trust across stakeholders |
| 1. Attitudes | Barrier: mixed acceptability amongst clinicians, scepticism about VWs reducing demand on NHS  Facilitator: high levels of enthusiasm of participants towards VWs |
| D. Motivation | Barriers: perceived as extra work, top-down pressure reduces intrinsic motivation  Facilitator: some clinicians highly motivated |
| **Patient and carers (innovation recipients)** | |
| A. Need | Barrier: belief that sometimes hospital care is in patients’ best interests  Facilitator: belief that care at hone is in patients’ best interests |
| B. Capability | Individual differences in digital competence |
| 1. Attitudes | Facilitator: General preference to receiving care at home |
| C. Opportunity | Barrier: Inequalities affect access to digital services and living situations |
| D. Motivation | Barrier: Some patients reject offer of VWs, not all carers support VWs  Facilitator: some carers support care at home |
| **V. IMPLEMENTATION PROCESS DOMAIN** | |
| A. Teaming | Collaboration strengthened over time, with multidisciplinary teams, steering groups, and broader partnerships, leading to a more unified approach by TP2. |
| B. Assessing Needs | |
| 1. Innovation Deliverers | Formal methods like surveys and workshops were rarely used to assess deliverers' needs. Feedback was mostly gathered informally, influencing decisions such as investing in community care capacity. |
| 2. Innovation Recipients | Some methods of capturing patient experience described (e.g. case studies). One participant discussed a wider movement towards patients possessing more autonomy over their own care through access to digital solutions, indicating a readiness of patients to engage in VWs. |
| C. Assessing Context | A minority of participants described minimal examples of reflecting on the barriers and facilitators to implementing VWs, including mapping out existing services at TP1, and quarterly case-based learning at TP2. |
| D. Planning | While initial project timelines and bed targets were required for funding approval by NHSE, there was little long-term, detailed planning due to financial uncertainty and limited knowledge of VW implementation. Scoping existing pathways was more common than defining specific roles and processes. Planning was mostly iterative, refining implementation over time rather than being fully developed beforehand. By TP2, planning efforts included social care audits to assess VWs' impact. The approach varied across ICSs, with some prioritising digital monitoring systems first and others focusing on clinical pathways. |
| E. Tailoring Strategies | Implementation varied across ICSs, adapting to barriers through strategies like innovative recruitment, case-based learning for clinical concerns, and dedicated staff for interoperability and procurement issues. |
| F. Engaging | |
| 1. Clinical staff (Innovation Deliverers) | Participants described a range of activities aimed at increasing buy-in from staff. |
| 2. Patients/Carers (Innovation Recipients) | It was evident that feedback from patients had informed subsequent implementation and delivery of VWs. Some participants also noted the importance of partnering with patients to engage them in VWs, as well as communication strategies and renaming VWs to ‘hospital at home’. |
| G. Doing | There were various references to starting cautiously, including ‘starting small’, ‘testing’, a ‘soft launch’ and a ‘phased approach’, particularly at TP2. Participants recognised that this approach allowed them to troubleshoot and refine. However, many participants utilised existing systems and pathways to implement VWs, thus ‘baby steps’ (P15, TP2) were less relevant and appropriate. |
| H. Reflecting & Evaluating | |
| 1. Implementation | Success indicators for Virtual Wards (VWs) included patient referrals, digital solution usage, clinician buy-in, project longevity, integration into routine care, and staff capacity. However, details on data collection methods were not provided. |
| 2. Innovation | Participants emphasised the need for a funded, systematic evaluation of Virtual Wards (VWs) to assess their costs and benefits. Evaluation focused on both quantitative and qualitative data, with some developing formal ‘evaluation plans’ to measure outcomes such as hospital admission rates, length of stay, emergency visits, patient satisfaction, and digital solution feedback. By TP2, particularly in areas with more established VWs, participants referenced findings from formal evaluations and routine data analysis, including modelling. |
| I. Adapting | At TP2, participants cited specific examples of how settings worked to overcome the pronounced barrier of the lack of interoperability between digital solutions utilised by VWs and existing digital solutions. Examples included choosing technology providers with proven integration with the clinical record system, developing a dashboard to combine information from several systems, and utilising existing technological systems and pathways. |
| **CFIR OUTCOMES ADDENDUM** | |
| **I. ANTECEDENT ASSESSMENTS** | |
| A. Acceptability | Those who had conducted evaluative work and gained patient feedback found patient acceptability to be much higher than anticipated, as many preferred to receive care in their own home. There were some safety concerns amongst patients particularly regarding if they become ill at night but mostly the lowest acceptability was amongst clinicians. |
| B. Appropriateness | Participants expressed concerns about the differential appropriateness of VWs for different patient groups, and the balance of optimising the inclusivity of VWs whilst ensuring that VWs are appropriate for patients’ needs. |
| C. Feasibility | Perceived feasibility of VWs was highly dependent on barriers to implementation, and at TP2 it became apparent that VWs were not feasible for all patient groups including palliative care. |
| D. Implementation Climate | Participants discussed a general resistance to change worsened by high workload pressure but reduced by cohesion between leadership and management and allowing for ownership of new innovations through adaptation. |
| E. Implementation Readiness | Across timepoints, readiness was higher when the implementation of VWs was built upon existing similar services and systems and their associated relationships and infrastructure, which often indicated maturity of ICSs. |
| **II. IMPLEMENTATION OUTCOMES** | |
| A. Anticipated Implementation Outcomes | |
| 1. Adoptability | There was a strong top-down enforcement of VWs given political agendas, thus implementation was expected to continue and expand. |
| 2. Implementability | In practice, logistical unanticipated issues due to lack of planning and sustainable funding meant implementation could be slower than expected. |
| 3. Sustainability | Participants recognised the potential for VWs to be a sustainable model of care delivery, dependent on continued funding. |
| B. Actual Implementation Outcomes | |
| 1. Adoption | Implementation of VWs was building traction at TP2 but varied across sites due to factors including clinician buy-in and limited staff capacity. |
| 2. Implementation | AT TP2, implementation outcomes diverged; some sites scaled back implementation due to lack of success, whilst others scaled-up. |
| 3. Sustainment | At TP2, utilisation of existing infrastructures and processes was noted to ensure sustainability of VWs. |
| **III. INNOVATION OUTCOMES** | |
| A. Innovation Recipient Impact | Some participants reported positive outcomes including patient satisfaction. |
| B. Innovation Deliverer Impact | Some participants noted reduced winter pressures and positive feedback from staff. |
| C. Key-Decision Maker (or System) Impact | Some participants noted prevention of admissions. |

Note: Domains were removed from the framework wherever there was no data coded within it.
